# Novel Natural Product- and Privileged Scaffold-Based Tubulin Inhibitors Targeting the Colchicine Binding Site

**DOI:** 10.3390/molecules21101375

**Published:** 2016-10-15

**Authors:** Mengqi Dong, Fang Liu, Hongyu Zhou, Shumei Zhai, Bing Yan

**Affiliations:** 1 School of Chemistry and Chemical Engineering, Shandong University, Jinan 250100, China; dongmengqi029@163.com (M.D.); liufanghuaxue@163.com (F.L.); 2 School of Environment, Guangzhou Key Laboratory of Environmental Exposure and Health and Guangdong Key Laboratory of Environmental Pollution and Health, Jinan University, Guangzhou 510632, China; hyzhou001@gmail.com

**Keywords:** tubulin inhibitors, microtubule dynamics, antimitotic, multidrug resistance

## Abstract

Tubulin inhibitors are effective anticancer agents, however, there are many limitations to the use of available tubulin inhibitors in the clinic, such as multidrug resistance, severe side-effects, and generally poor bioavailability. Thus, there is a constant need to search for novel tubulin inhibitors that can overcome these limitations. Natural product and privileged structures targeting tubulin have promoted the discovery and optimization of tubulin inhibitors. This review will focus on novel tubulin inhibitors derived from natural products and privileged structures targeting the colchicine binding site on tubulin.

## 1. Introduction

Microtubules, a key component of the cytoskeleton, play essential roles in important cellular events, such as the maintenance of cell shape, cell division, cell migration and intracellular transport. Microtubules are dynamic polymers composed of tubulin heterodimers formed from α- and β-tubulins. The dynamics of microtubule polymerization are tightly regulated. Under normal physiological conditions, a dynamic equilibrium exists between the intracellular pool of α,β-tubulin heterodimers and the microtubule polymer [1,2]. The dynamics of microtubule polymerization are central to their biological functions.

In dividing cells, the most important role of microtubules is to form the mitotic spindle involved in cell division. This function makes microtubules an attractive target for anticancer therapy. To date, many chemically diverse compounds have been discovered and shown to possess anticancer activities by binding to tubulin and inhibiting the functions or dynamics of microtubules. These compounds are known as antimitotic agents and include taxanes, paclitaxel, vincristine, colchicine, docetaxel, ixabepilone, cabazitaxel and navelbine [3]. Based on their effects on microtubule polymerization, antimitotic agents are divided into two major classes [4]: microtubule-destabilizing agents and microtubule-stabilizing agents. The destabilizing agents, such as vinca alkaloids and colchicine, inhibit tubulin polymerization by binding to the vinca domain or the colchicine domain (at the α-β interfacial site) of tubulin. The microtubule-stabilizing agents, including taxanes, paclitaxel, docetaxel and epothilones, stimulate tubulin polymerization by binding to an overlapping taxoid binding site on β-tubulin, which is located on the inner surface of the microtubule [5]. The well-characterized binding sites for these agents on tubulin include the taxoid binding site, the vinca site, and the colchicine site [2,3].

The binding of antitubulin agents regulates microtubule polymerization at high concentrations or suppresses microtubule dynamics at lower concentrations (10–100 times lower), which is the common mechanism by which these drugs block mitosis and induce cell apoptosis [6,7]. Other mechanisms of action include anti-angiogenesis activity and vascular disruption [3]. Over the last two decades, much effort has been devoted to identifying structurally diverse inhibitors of tubulin polymerization from both natural and synthetic sources. Notably, most of the known active antitubulin agents are of natural origin, characterized by a very complex structure and are difficult to synthesize and scale up. Other drawbacks of the use of tubulin inhibitors in cancer therapy include multidrug resistance (MDR), dose-limiting toxicity and poor water solubility. Thus, many studies have been performed over the years to explore new antitubulin agents inspired by natural products, which include combretastatin A-4, chalcones, phenstatins, podophyllotoxin, chalcones, taxol and vinca alkaloids. Structurally, these small active molecules have various heteroaromatic cores, such as the indole [8], benzofuran [9,10], thiazole [11,12] and imidazole [13,14,15] moieties. For many years, the potential of using colchicine binding site inhibitors (CBSIs) to overcome the clinical limitations of the available tubulin inhibitors has been recognized [16]. In this review, we will focus on novel, chemically diverse antitubulin agents targeting the colchicine binding site on tubulin.

## 2. Colchicine Analogues

**Figure 1:**
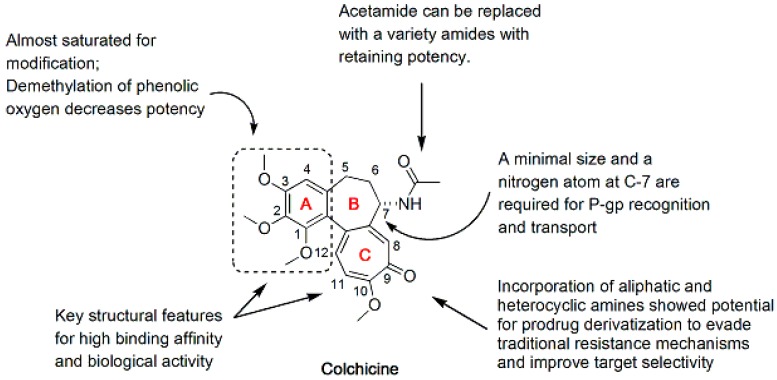
SAR of colchicine.

Colchicine (**1**), a natural product extracted from *Colchicum autumnale*, has been used to treat acute gout. Although colchicine (**1**) is a powerful antimitotic agent, the use of colchicine in cancer chemotherapy is hampered by its toxicity and the development of MDR [17]. However, the remarkable biological activity of colchicine in microtubule depolymerization has stimulated the exploration of new colchicine analogues with improved anticancer activity and low systemic toxicity. Structurally, colchicine consists of a trimethoxyphenyl ring (A-ring), a saturated seven-membered ring containing an acetamido group (B-ring), and a tropolone ring (C-ring) (Figure 1). As shown in previous structure-activity relationship (SAR) studies, the A- and C-rings of colchicine are key structural features required for its high binding to tubulin and biological activity [18,19,20,21]. Any modification in the A-ring of colchicine causes a complete loss of binding, whereas modifications to both the B- and C-rings are possible. In addition, both a minimal size and a nitrogen atom at C-7 of the B-ring are required for P-glycoprotein (P-gp) recognition and transport [22] (Figure 1). Therefore, many studies have focused on structural modifications of C-7 of the B-ring to discover colchicine analogues with improved potency and less toxicity or analogues that overcome drug resistance.

**Figure 2:**
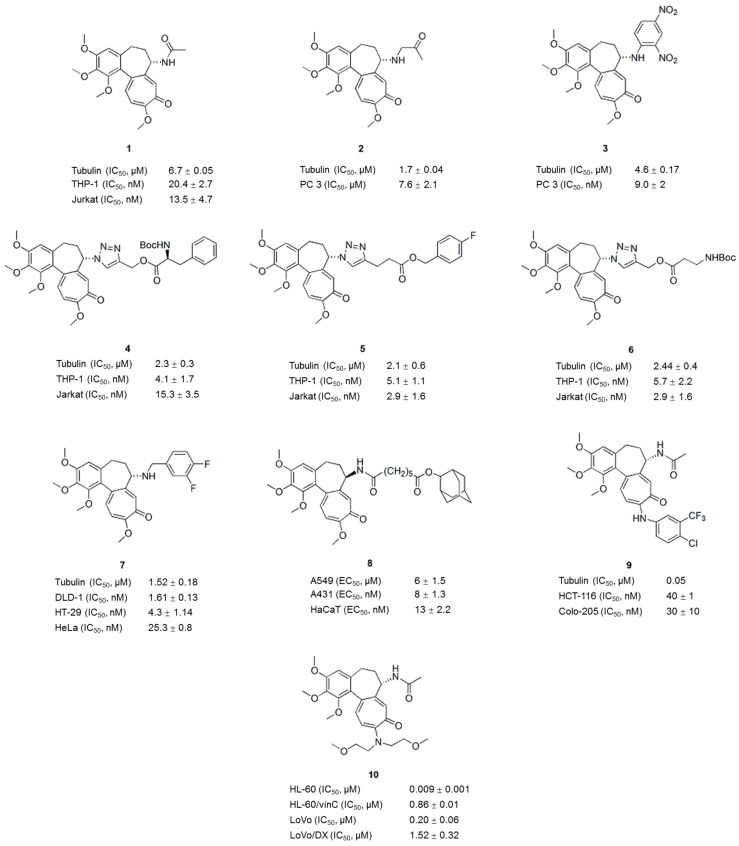
Structures of colchicine derivatives.

For example, colchicine and isocolchicine analogues modified at the C-7 position of the B-ring have been reported [23]. In addition to the strong inhibition of tubulin polymerization, compounds **2** and **3** (Figure 2) also possessed an increased cytotoxic activity compared to that of colchicine. Among the new colchicinoids with a variable triazole unit at C-7 [24], three active compounds, the phenylalanine derivative **4** (Figure 2), the 4-fluorobenzyl ester **5** (Figure 2), and the β-alanine ester **6** (Figure 2), exhibited pronounced cytotoxic activities against several human cancer cell lines and inhibited tubulin polymerization, with IC_50_ values ranging from 2–3 μM. These active compounds distorted the microtubule morphology by depolymerizing tubulin; in particular, compound **4** (Figure 2), also exhibited a pronounced centrosome declustering effect in triple negative breast cancer cells (MDA-MB-231) and non-small cell lung cancer cells (H1975). Based on the SAR analysis, the distance between the lipophilic moiety (phenyl or tert-butyl) and the colchicine core in the colchicine-derived triazoles played a key role in improving their anticancer activities.

In another study, colchicine B-ring analogues were prepared by incorporating aryl-substituted fragments to improve the affinity and to overcome MDR [25]. Compared with colchicine, compound **7** (Figure 2) possesses improved antiproliferative activity against drug-sensitive (IC_50_ < 30 nM) and drug-resistant cancer cells (IC_50_ values for A2780A and HeLa*β*III cells are 17.2 ± 1.4 and 14.1 ± 0.8 nM, respectively) and inhibited tubulin polymerization (IC_50_: 1.52 ± 0.18 μM). Furthermore, colchicine analogues modified at the C-ring through the incorporation of aliphatic and heterocyclic amines showed potential for prodrug derivatization to evade traditional resistance mechanisms and improve target selectivity [26]. In the study by Zefirova et al., several colchicine analogues with various modifications at C-7 of the B-ring not only showed a colchicine-like ability to promote microtubule disassembly but also induced tubulin assembly into clusters [27,28]. The active compounds, which inhibited the growth of the human lung carcinoma cell line A549 at nanomolar concentrations (EC_50_ < 13 nM), were several times more cytotoxic than colchicine and exhibited both microtubule depolymerizing and tubulin clustering activities. Based on the SAR analysis of the analogues, both cytotoxicity and tubulin clustering ability were very sensitive to the linker length but were less sensitive to the position of the linker. The presence of a bulky hydrophobic and non-aromatic moiety in compound **8** (Figure 2) played an important role in the formation of tubulin clusters. Compound **8** (Figure 2) competed with both colchicine and taxol for binding to tubulin in vitro and induced tubulin assembly into regular wavy-shaped clusters composed of protofilaments bundles [28]. The proposed mechanism of compound **8** and its analogues includes specific interactions with the α-tubulin subunit and the induction of cell cycle arrest at G_2_/M and apoptosis.

Recently, new colchicine derivatives with C- and B-ring substitutions were evaluated for P-gp induction and antiproliferative activity [29]. A number of derivatives showed a lower P-gp-induction liability compared to colchicine due to their reduced ability to interact with and change the conformation of P-gp. The most active compound, **9** (Figure 2), showed antiproliferative activity in HCT-116 cells (IC_50_: 0.04 μM) and inhibited microtubule assembly and tumor growth in a mouse model, revealing new opportunities for the colchicine scaffold. Amine derivatives of colchicine have been obtained by modifying the C(10)-OCH_3_ position of the C-ring and evaluating their cytotoxicity against drug-sensitive and drug-resistant cancer cell lines [30]. Among these compounds, compound **10** (Figure 2) with a bis(2-methoxyethyl)amine substituent is the most active compound, with IC_50_ in the nanomolar to submicromolar concentrations, indicating the importance of the amine substitution at the C-10 position of the C-ring for anticancer activity and selectivity. In addition, hybrid molecules containing the colchicine moiety and a pironetin analogue fragment connected through an ester-amide spacer of variable length have been prepared [31]. These compounds showed lower cytotoxicity than the parent molecules colchicine and pironetin, and the length of the connecting spacer influenced the binding of the hybrid compounds to tubulin. Hybrid molecules with short spacers will reversibly bind the colchicine site and molecules with longer spacers covalently bind the pironetin binding site. In conclusion, appropriate modification of colchicine or hybridization with other active molecules can attenuate toxicity, which is of great benefit in cancer treatment.

## 3. Combretastatin A-4 Analogues

**Figure 3:**
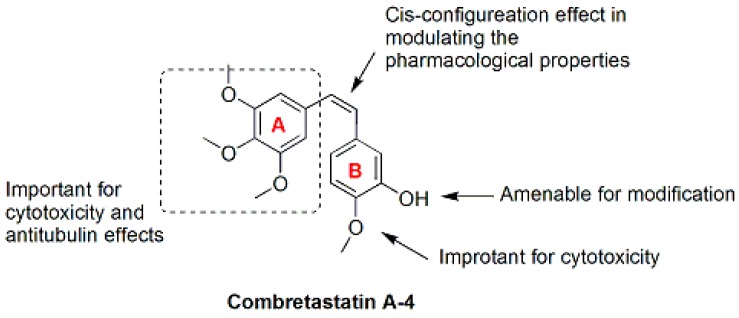
SAR of CA-4 [15].

**Figure 4:**
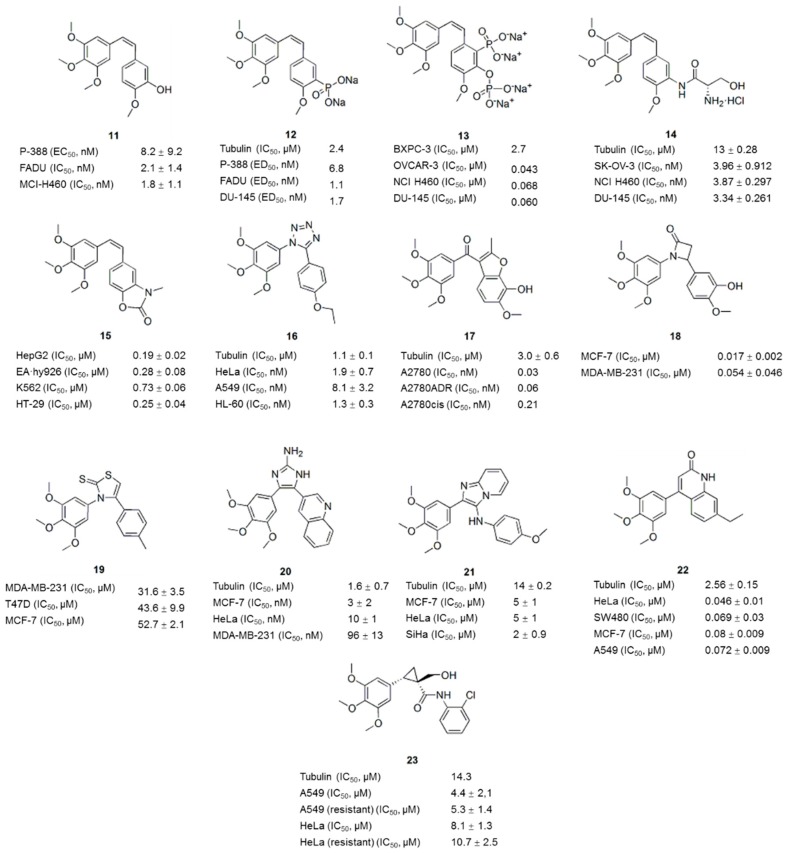
Combretastatin A-4 analogues.

Combretastatin A-4 (CA-4, **11**, Figure 3 and Figure 4), a natural *cis*-stilbene derivative isolated from the bark of the African willow tree *Combretum caffrum*, is a typical inhibitor of tubulin polymerization that binds to the colchicine binding site [32]. CA-4 shows strong cytotoxicity against a variety of cancer cells, including MDR cell lines [33]. However, poor solubility and the isomerization to the less active *trans*-form prevent the clinical translation of CA-4 [34]. The SAR (Figure 3) analysis showed that trimethoxy moiety, the *cis*-configuration of the double bond and the *para*-methoxy group on the B-ring exert dominant effects on modulating the pharmacological properties [35,36]. Because modifications of the A-ring reduce the bioactivity of most CA-4 (**11**) analogues (Figure 4), other studies have focused on modifying the B-ring and the bridge [35,37]. The analogues CA4P (**12**) (Figure 4), CA-1P (OXi4503: **13**) (Figure 4) and AC7700 (AVE8062: **14**) (Figure 4), three early prodrugs of CA-4 obtained by modifying the B-ring, are currently in clinical trials as potential vascular-targeting agents [38,39,40,41,42,43]. Currently, a large number of analogues of CA-4 (**11**) has been evaluated, some of which have recently been reviewed elsewhere [33].

### 3.1. B-Ring-Modified Analogues

Modifications to the B-ring are further subdivided into three main lines of research: (a) substituted phenyl rings, (b) heterocyclic rings, and (c) non-substituted aromatic rings. As shown in the SAR analysis, the presence of a *para*-methoxy group was required for the biological activity, whereas the presence of the *meta*-hydroxyl group was not essential. The *meta* position can undergo substitution with electron-donating (e.g., amino) [35] or electron-withdrawing groups (e.g., fluorine) [37], but substituents cannot exceed the size of the hydroxyl group. Among those analogues, heterocyclic rings were often exchanged for the benzene B-ring. Thus, compound libraries were prepared with indole [44], benzoxazolone [45], benzothiophene [46] and naphthalene [47] as the B-rings. These analogues maintained the ability to bind tubulin and inhibit microtubule polymerization. Furthermore, some modifications exhibited enhanced cytotoxicity. For example, compound **15** (Figure 4), one of the compounds prepared by replacing the B-ring with a benzoxazolone scaffold, showed enhanced cytotoxicity against several human cancer cell lines, including a combretastatin-resistant cell line, with IC_50_ values ranging from 0.19 to 0.73 μM [45].

### 3.2. Bridge-Modified Analogues

The linkage was reported to tolerate modifications, but the *cis*-configuration is required for the high cytotoxicity and antitubulin activity. Successful modification of the double bond was mainly achieved by replacing the olefinic bridge with heterocyclic rings to maintain a *cis*-locked configuration [48,49]. Alternatively, because heterocyclic compounds usually possess pharmacological properties, these substitutions might improve the therapeutic potential of these agents. The substitution of the double bond with five-membered rings appears to be the best option [36]. Currently, heterocyclic moieties such as triazoles [49], tetrazoles [50], furan [10], β-lactam [51], thiazole [12], imidazole [15], and selenadiazole [52], thiophene [53] have been shown to maintain or enhance the biological activities. For example, the 1,2,3,4-tetrazole ring is a suitable mimic to identify potent antiproliferative agents and novel inhibitors of tubulin polymerization that act at the colchicine site [50]. Compound **16** (Figure 4) is a representative example of one such structure that exhibited anticancer activity in eleven cancer cell lines, including MDR cancer cells, with IC_50_ values below 10 nM; compound **16** also significantly reduced the growth of HT-29 xenografts in vivo. Compound **17** (Figure 4) was identified from a screen of a focused library of benzo[*b*]furans as a potent inhibitor of tubulin polymerization (IC_50_: 3.0 ± 0.6 μM) with selectivity toward activated endothelial cells and a panel of cancer cell lines, including a range of MDR cells [10]. The disodium phosphate ester prodrug of compound **17** exhibited superior vascular-disrupting and tumor growth inhibitory properties compared with CA4P (**12**).

The incorporation of the 1,4-diaryl-2-azetidinone (β-lactam) ring system in place of the ethylene bridge resulted in CA-4 analogues with potent antiproliferative activity in breast cancer cells [51]. Among these compounds, the most potent compound **18** (Figure 4), exhibited low nanomolar activity in both MCF-7 and MDA-MB-231 cells and inhibited the tubulin polymerization with improved efficacy compared with CA-4 (**11**). Compound **19** (Figure 4), in which the olefinic core structure of CA-4 (**11**) was replaced with 2(3*H*)-thiazole thione, selectively inhibited cancer cell proliferation (IC_50_: 31.6–52.7 μM) and depolymerized tubulin [12].

Compound **20** (Figure 4), one of the 4,5-biarylated-2-aminoimidazole CA-4 analogues, showed higher antiproliferative activity than CA-4 (**11**) against five different cancer cell lines, including a drug-resistant cell line, and had an IC_50_ value of less than 3 nM [15]. Compound **20** inhibited the assembly of purified tubulin with an IC_50_ of 1.6 μM and strongly perturbed the microtubule network. Sanghai et al. synthesized a series of novel 2-aryl-3-arylamino-imidazo-pyridines/pyrazines [54]. Among these compounds, compound **21** (Figure 4) significantly inhibited tubulin polymerization and disrupted tubulin-microtubule dynamics, similar to CA-4 (**11**). These compounds showed potent anticancer activities in kidney, breast and cervical cancer cell lines and relatively low toxicity to normal cells compared to CA-4 (**11**). As shown in a molecular modeling analysis, the bridging NH and the imidazo-pyridine/pyrazine ring in the title compounds provided additional pharmacophoric features for the inhibition of tubulin polymerization. In addition, the hexa-cyclic compounds bearing B-C ring condensation and containing a C=C bond in the B-ring (**22**) (Figure 4) exhibited excellent antiproliferative activities against various cancer cell lines at nanomolar concentrations (IC_50_: 46–80 nM) and inhibited tubulin assembly at micromolar concentrations (IC_50_: 2.56 ± 0.15 μM) [55]. Instead of inhibiting tubulin polymerization, novel cyclopropylamide analogues of CA-4 derivatives were able to stimulate tubulin polymerization [56]. Compound **23** (Figure 4), the most potent analogue, showed antiproliferative activity against seven human cancer cell lines, including paclitaxel-resistant cancer cells (IC_50_: 2~20 μM), and effectively inhibited tumor growth in the A549 xenograft model without causing significant body weight loss. In the analysis of pyridine-bridged analogues of CA-4 (**11**) reported by Zheng et al., the activities of the analogues with 3-atom linkers varied widely, depending on the phenyl ring substitutions, but the most potent compounds exclusively display the nitrogen-containing linker configuration [57].

## 4. Compounds with an Indole Nucleus

The indole skeleton is a scaffold that probably represents one of the most important structural subunits for the discovery of new drug candidates [58,59]. A number of tubulin polymerization inhibitors, such as arylthioindoles, aroylindoles, diarylindoles and indolylglyoxyamides, include an indole nucleus [8,60,61].

**Figure 5:**
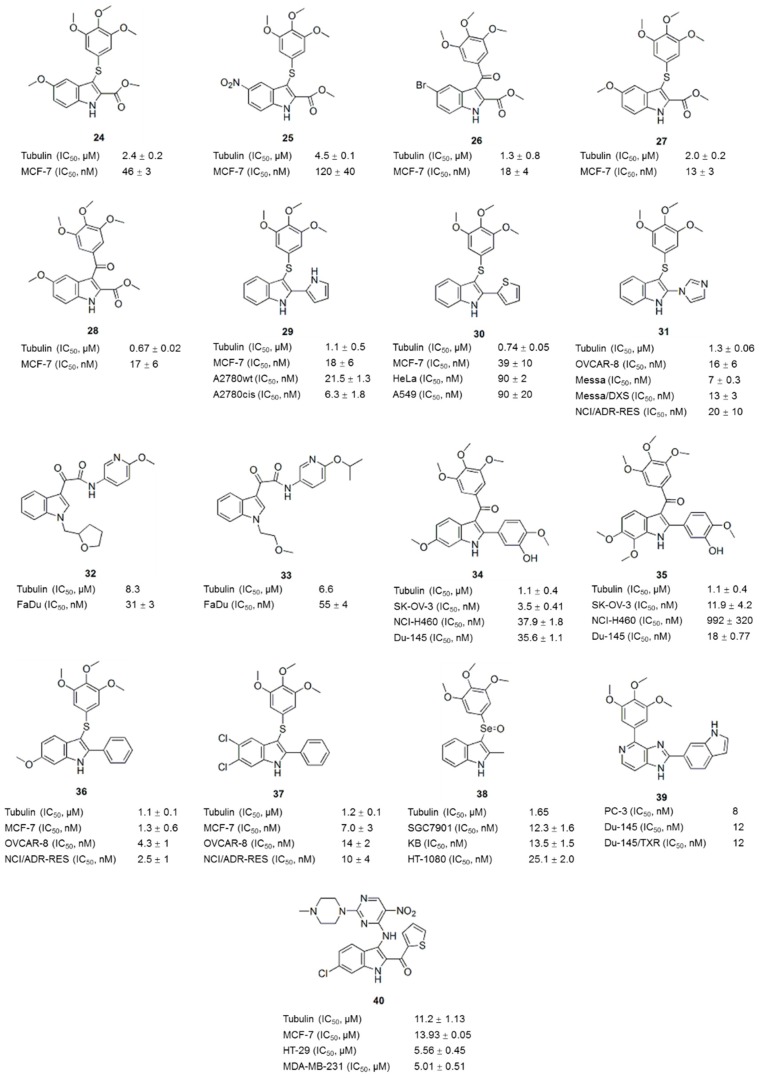
Arylthioindole derivatives and other indole-based compounds.

Arylthioindoles (ATIs) are a class of potent inhibitors of tubulin polymerization and cancer cell growth [62]. The ATIs inhibit tubulin assembly by interacting with the colchicine site on β-tubulin [8]. In recent years, excellent work on the optimization of the structures of ATI derivatives and SAR analyses has been reported [62,63,64,65,66,67]. For example, compounds **24** (Figure 5) and **25** (Figure 5) inhibited tubulin polymerization with IC_50_ values of 2.0 and 4.5 μM, respectively, and inhibited MCF-7 cell growth at nanomolar concentrations [62,63]. Some ATI derivatives bearing a halogen atom or a small-sized ether group at position 5 of the indole moiety also inhibited tubulin polymerization to a comparable level as colchicine (**1**) and CA-4 (**11**) [64]. ATI derivatives, along with the corresponding ketone and methylene compounds, were potent inhibitors of tubulin assembly with IC_50_ values ranging from 0.67 to 4.6 μM [65], and some compounds (**26**, **27** and **28**) (Figure 5) inhibited human MCF-7, HeLa and HCT116/chr3 cell growth by 50% at nanomolar concentrations. ATI derivatives in which the 2-alkoxycarbonyl group was replaced with a bioisosteric 5-membered heterocycle, such as a pyrrole, furan, or thiophene moiety, not only inhibited tubulin polymerization and cancer cell growth but also exhibited high metabolic stability (**29**, **30**) (Figure 5) [66]. ATI derivatives possessing different cyclic substituents at position 2 of the indole inhibited tubulin polymerization with IC_50_ values ranging from <1.0 to 2.0 μM and inhibited the growth of MCF-7 cells, with IC_50_ values in the nanomolar range [67]. The most potent compound **31** (Figure 5), was uniformly active in a larger panel of cancer cells, including MDR cells, and showed initial vascular-disrupting effects in a tumor model of liver rhabdomyosarcoma [67].

In addition to the ATI derivatives, other potential tubulin inhibitors containing an indole ring as the core nucleus have also been investigated. For example, compounds (**32**, **33**) (Figure 5) designed based on indole-3-glyoxylamides inhibited tubulin polymerization (IC_50_ values of 8.3 and 6.6 μM, respectively) and displayed improved cancer cell cytotoxicity in vitro (LC_50_ values of 31 and 55 nM against the FaDu cell line, respectively) and in vivo [68]. Inspired by OXi8006 (**34**, Figure 5), a well-known 2-aryl-3-aroyl indole-based potential tubulin inhibitor, a series of diversely functionalized analogues were prepared for biological evaluation. Compound **35** (Figure 5), which incorporates methoxy group at position 7, showed comparable activity in terms of the inhibition of tubulin assembly (IC_50_ < 5 μM) and cytotoxicity against three human cancer cell lines (SK-OV-3, NCI-H460 and DU-145) [69]. Two representative members of the 2-phenylindole derivatives of the indole (compounds **36** and **37**, Figure 5) inhibited tubulin polymerization with IC_50_ values of 1.0–2.0 μM, and uniformly inhibited a panel of cancer cells at nanomolar concentrations [70]. However, the replacement of the B-ring and the *cis* olefinic core of CA-4 (**11**) with an indole moiety and selenium atom, respectively, maintained or slightly improved the antiproliferative activity of the compound [71]. Compound **38** (Figure 5), the most potent derivative, inhibited the proliferation of three human cancer cell lines (SGC7901, KB and HT-1080) with IC_50_ values of 12.3 ± 1.6, 13.5 ± 1.5 and 25.1 ± 2.0 nM, respectively. Compound **38** inhibited tubulin polymerization and disrupted microtubule dynamics in a similar manner to CA-4 (**11**).

In recent years, a series of novel hybrids of two crucial components of the pharmacophore in antitubulin drugs have resulted in many antitubulin agents with improved activity compared to the parent structures. For example, Kamal et al. [72] synthesized imidazopyrimidine-oxindole conjugates based on the biological activities of oxindole and imidazopyridine moieties. Some of the compounds displayed impressive antiproliferative activities (GI_50_: 0.17–9.31 μM) against sixty different human cancer cell lines and remarkable inhibitory effects on tubulin polymerization, similar to CA-4 (**11**). Hwang et al. synthesized a series of indolyl-imidazopyridines (IIP) and found that some potently inhibited tubulin polymerization in a panel of human melanoma and prostate cancer cell lines, with IC_50_ values ranging from 3 to 175 nM [73]. The 6-indolyl derivative **39** (Figure 5) showed the strongest inhibition (IC_50_ at 3 nM on A375 and 8 nM on PC-3) and best metabolic stability, 56.3 min, in human liver microsomes (HLM). As shown in the SAR analysis, the pyridine D-ring moiety of IIP provided some benefits toward metabolic stability in HLM. Hu et al. synthesized novel hybrids of an indole-pyrimidine containing a piperazine moiety [74]. The majority of these compounds possessed significant cytotoxicity. The most promising compound **40** (Figure 5) inhibited tubulin polymerization (IC_50_: 11.2 μM) and showed broad-spectrum cytotoxicity (IC_50_ values ranged from 5.01 to 14.36 μM) toward several human cancer cell lines, but not normal human cells. Thus, hybrids of pharmacophores might be an efficient strategy for the development of tubulin inhibitors.

## 5. Chalcone Analogues

**Figure 6:**
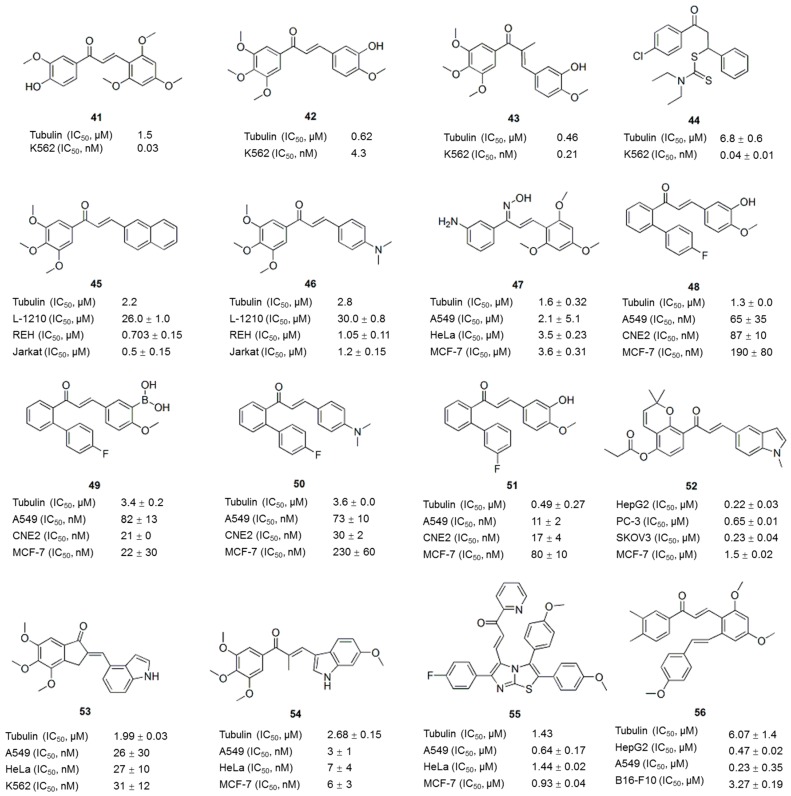
Chalcone analogues.

Chalcones, the precursors of flavonoids and isoflavonoids, are abundant in edible plants. Chalcone comprises a characteristic framework of 1,3-diaryl-2-propen-1-one and represents an attractive scaffold for the design of novel colchicine site ligands that inhibit tubulin assembly [75]. As chalcones are easy to synthesize and contain excellent leading skeletons, studies have been performed to modify chalcones and enhance their antitumor activities [76]. For example, Lawrence et al. reported the synthesis of a 644-membered library of chalcones by parallel synthesis using the Claisen-Schmidt reaction [77]. Seven chalcones exhibited an IC_50_ of less than 1 μM against K562 cells. The most active compound **41** (Figure 6) was impressively cytotoxic (IC_50_: 30 nM) and inhibited tubulin polymerization (IC_50_: 1.5 μM) at levels comparable to CA-4 (**11**). Ducki et al. incorporated the aryl substitution pattern of CA-4 (**11**) into chalcones and obtained several chalcones with substantial in vitro activity against the K562 human leukemia cell line [78]. As shown in the SAR analysis, the 3,4,5-trimethoxy A-ring substitution markedly increases cytotoxicity, and the most cytotoxic analogues are those chalcones most resembling CA-4 (**11**), such as compounds **42** and **43** (Figure 6). Compound **44** (Figure 6), the most potent compound of a series of novel dithiocarbamate compounds with the chalcone scaffold [79], inhibited the growth of MCF-7 cells with an IC_50_ value of 0.04 ± 0.01 μM, and tubulin polymerization, with an IC_50_ value of 6.8 ± 0.6 μM. Compounds **45** and **46** (Figure 6) were obtained from a series of trimethoxychalcones [80]. These compound inhibited tubulin assembly similar to colchicine (**1**), with IC_50_ values of 2.2 μM and 2.8 μM, respectively, and selectively inhibited the growth of various human cancer cell lines at nanomolar concentrations, causing microtubule destabilization and mitotic arrest. The SAR analysis suggested that the presence of a 3,4,5-trimethoxyphenyl group in the A-ring is beneficial for tubulin interaction as well as antiproliferative and antimitotic effects [80]. As reported by Wang et al., chalcone oxime derivatives maintained their ability to inhibit tubulin polymerization and the antiproliferative activity against human cancer cell lines by binding to the colchicine binding site [81]. Among these novel compounds, compound **47** (Figure 6) displayed the most potent inhibition of tubulin polymerization (IC_50_: 1.6 μM) and displayed potent antiproliferative activity against A549, HeLa and MCF-7 cells, with GI_50_ values of 2.1, 3.5 and 3.6 μM, respectively, which were comparable to colchicine (**1**) and CA-4 (**11**).

Compounds **48**, **49**, **50** and **51** (Figure 6) are *ortho*-aryl chalcones. These compounds inhibited microtubule polymerization and exhibited significant antiproliferative activity against a panel of parental and MDR cancer cell lines with IC_50_ values ranging from moderate to low nanomolar concentrations. This activity is several thousand-fold superior to the abilities of colchicine, paclitaxel, doxorubicin, and vincristine to inhibit the growth of MDR cancer cell lines, implying that *ortho*-aryl chalcones represent a new scaffold targeting tubulin and mitosis for novel antitumor drug discovery [82]. Compound **52** (Figure 6) is a new pyranochalcone derivative containing an indole moiety. This compound displayed cytotoxic activity against ten human cancer cell lines, including cells with an MDR phenotype, with IC_50_ values ranging from 0.22 to 1.80 μM, and exerted potent anticancer activity against HepG2 human liver carcinoma in BALB/c nude mice [83]. An *N*-methyl-5-indolyl group on the right ring and a propionyloxy group at the 4 position of the left phenyl ring had beneficial effects on the cytotoxic activity. Chen et al. designed and synthesized a series of novel chalcone analogues through the aldol condensation of indanones and indole carbaldehydes [84]. Compounds obtained from 4,5,6-trimethoxy indanone with different indole carbaldehydes usually exhibited more potent antiproliferative activities. The GI_50_ values for the most potent compound, **53** (Figure 6), against five cancer cell lines ranged from 26 nM to 35 nM. Based on mechanistic studies, compound **53** effectively inhibited cellular tubulin polymerization and interfered with mitosis in vitro, resulting in a prolonged arrest of the cell cycle at G_2_/M and ultimately the apoptosis of cancer cells. Twenty-nine novel indole-chalcone derivatives were synthesized to develop additional tubulin inhibitors [85], among which compound **54** inhibited tubulin polymerization (IC_50_: 2.68 ± 0.15 μM) and exhibited the most potent activity against six cancer cell lines (IC_50_ values of 3–9 nM), with a 3.8- to 8.7-fold increase in activity compared with that of compound **53** (Figure 6). Compound **54** (Figure 6) displayed nearly equally potent antiproliferative activity toward drug-resistant cancer cell lines, good metabolic stability in mouse liver microsomes and antitumor activity in vivo without apparent toxicity, which was better than compounds CA-4P (**12**) and **53**.

In addition to individual compound, a number of potent hybrid molecules with anticancer activities have been developed by grouping different pharmacophores [86]. For example, the hybrid of imidazothiazoles and chalcones resulted in 2,3-diaryl imidazo[1,2-*b*]thiazole-chalcone conjugates that exhibited significant antiproliferative activities against several cancer cells. Among these conjugates, compound **55** (Figure 6), which contains a pyridyl ring, is the most active, with IC_50_ values ranging from 0.64 to 1.44 μM in all tested cell lines. This compound disrupted microtubule dynamics and induced the formation of an abnormal spindle structure and centrosome by binding tubulin at the colchicine site [87]. In another study, twenty-three compounds containing a resveratrol skeleton and a chalcone moiety were synthesized. Compound **56** (Figure 6) was the most potent compound in a cytotoxicity assay, and it inhibited the growth of the cells lines HepG2, B16-F10, and A549, with IC_50_ values of 0.47 ± 0.02, 3.27 ± 0.19, and 0.23 ± 0.35 μM, respectively, and inhibited tubulin polymerization, with an IC_50_ of 6.07 ± 1.4 μM [88].

## 6. Podophyllotoxin Derivatives/Analogues

**Figure 7:**
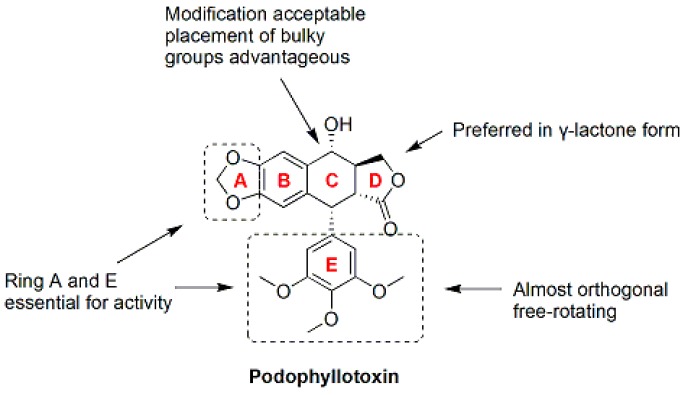
SAR of podophyllotoxin [94].

**Figure 8:**
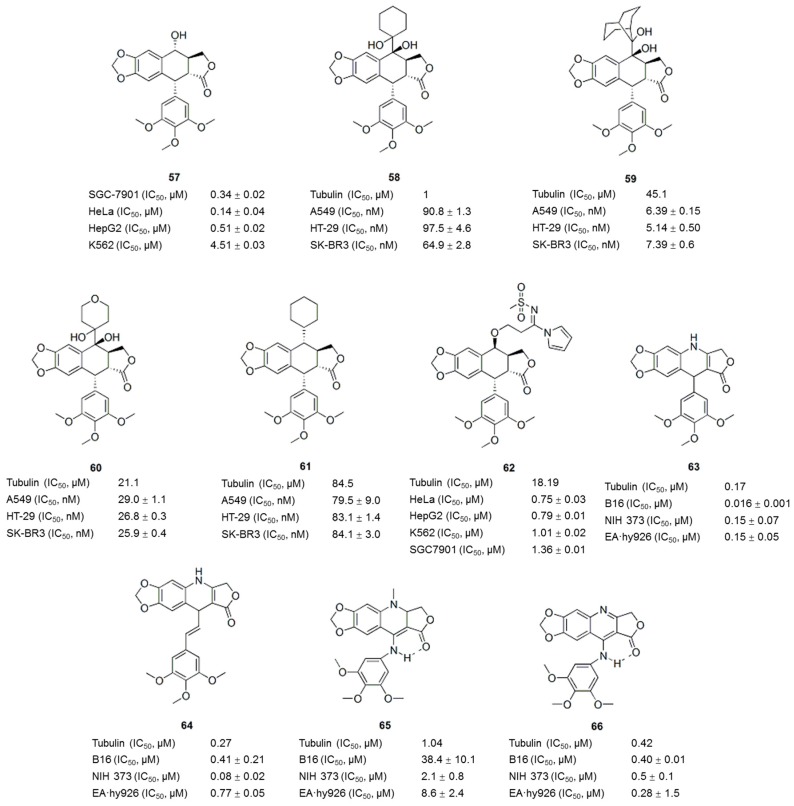
Podophyllotoxin and its derivatives/analogues.

Podophyllotoxin (**57**, PTOX, Figure 7 and Figure 8), a naturally occurring cyclolignan isolated from *Podophyllum* species, inhibits the assembly of tubulin into microtubules by binding to the colchicine site of tubulin [89]. The severe toxicity of PTOX limits its application as a cancer therapy. However, PTOX is still considered an attractive lead candidate for the development of new therapeutic agents [90]. Derivatives of podophyllotoxin, such as etoposide, etopophos and teniposide, have been developed and are currently used in the clinic for the treatment of a variety of malignancies. The main mechanism by which PTOX derivatives exert their antitumor activities is the inhibition of microtubule assembly and DNA topoisomerase II [89,90]. Podophyllotoxin-like compounds preferentially inhibit tubulin polymerization by reversibly binding to the colchicine site, which leads to arrest of the cell cycle in the metaphase [91]. Based on the structural complexity of podophyllotoxin arising from the presence of four stereogenic carbons in the C-ring, most of the SAR studies have been performed using podophyllotoxin analogues [92]. The structural features required for the antitubulin activity of podophyllotoxin analogues are the transfused γ-lactone, the fused dioxole ring, and the nearly orthogonal free-rotating 3,4,5-trimethoxyphenyl fragment [93] (Figure 7).

Recently, numerous modifications to podophyllotoxins structures have been generated to develop novel tubulin-targeting anti-tumor drugs. For example, novel pinacol derivatives of podophyllotoxins with different side chains at C-7 were synthesized through the reductive cross-coupling of podophyllotoxin with several aldehydes and ketones [93]. Analyses of the cytotoxicities toward neoplastic cells and the disruption of microtubules showed that epipodopinacol (7β-OH) analogues **58**–**60** (Figure 8) proved to be the most potent inhibitors of three cancer cell lines (A549, HT-29 and SK-BR3) with GI_50_ values less than 100 nM. Significantly, 7α-isopropyl-7-deoxy-podophyllotoxin (**61**, Figure 8), without any hydroxyl functional group, appeared to be as a promising lead compound for a novel type of tubulin polymerization inhibitor. Liu et al. [95] reported a biological evaluation of eight novel podophyllotoxin derivatives, of which compound **62** displayed significant antiproliferative activities against four human tumor cell lines, with IC_50_ values of 0.75–1.36 μM, as well as strong inhibition of tubulin polymerization. Labruere et al. synthesized two novel series of azapodophyllotoxin analogues. Several new compounds **63**–**66** (Figure 8) with inhibitory activity toward tubulin polymerization similar to CA-4 (**11**) and colchicine (**1**) were identified [96], indicating that 4-azapodophyllotoxin analogues are potential lead compounds for the development of vascular-disrupting agents.

## 7. Myoseverin Derivatives

**Figure 9:**
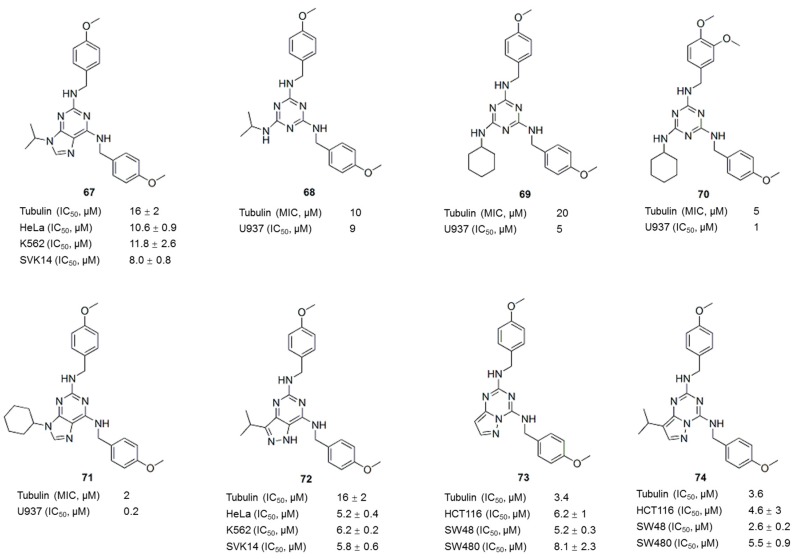
Myoseverin and its derivatives.

Myoseverin (**67**, Figure 9) was previously identified as a novel inhibitor of microtubule assembly from a screening of a 2,6,9-trisubstituted purine-based library [97]. The myoseverin-induced inhibition of microtubule assembly promoted the synthesis of novel trisubstituted triazine libraries [98,99,100]. Compounds identified from these libraries exhibit novel anti-tubulin activities in vitro and in vivo. For example, compounds **68** and **69** (Figure 9) exhibited activities comparable to myoseverin (**67**), whereas compound **70** (Figure 9) exhibited an at least 4- to 10-fold improvement in inhibition in tubulin and U937 cell growth assays compared to myoseverin (**67**) [98]. The N9 cyclohexyl derivative **71** (Figure 9) prevented microtubule polymerization in *Xenopus laevis* egg extracts and inhibited the polymerization of purified bovine brain tubulin [99]. The GI_50_ of compound **71** against U937 cells is 20-fold less than the GI_50_ of myoseverin (**67**). The SAR analysis showed that hydrophobic substituents at the N9 position potently inhibited microtubule assembly [99,101]. Further optimization of myoseverin led to the discovery of the pyrazolo[4,3-*d*]pyrimidine **72** (Figure 9) [100] and pyrazolo[1,5-*a*]-1,3,5-triazine (**73** and **74**) (Figure 9) [102] analogues that exhibited potent inhibition of tubulin polymerization and specific antiproliferative activity in cancer cell lines at micromolar concentrations. Compared with other agents interfere with microtubule, such as taxol, vinblastine, nocodazole and colchicine, the nontoxicity of these purine-based microtubule-interfering agents suggests that they are suitable for potential application as anticancer agents [101].

## 8. Sulfonamides

**Figure 10:**
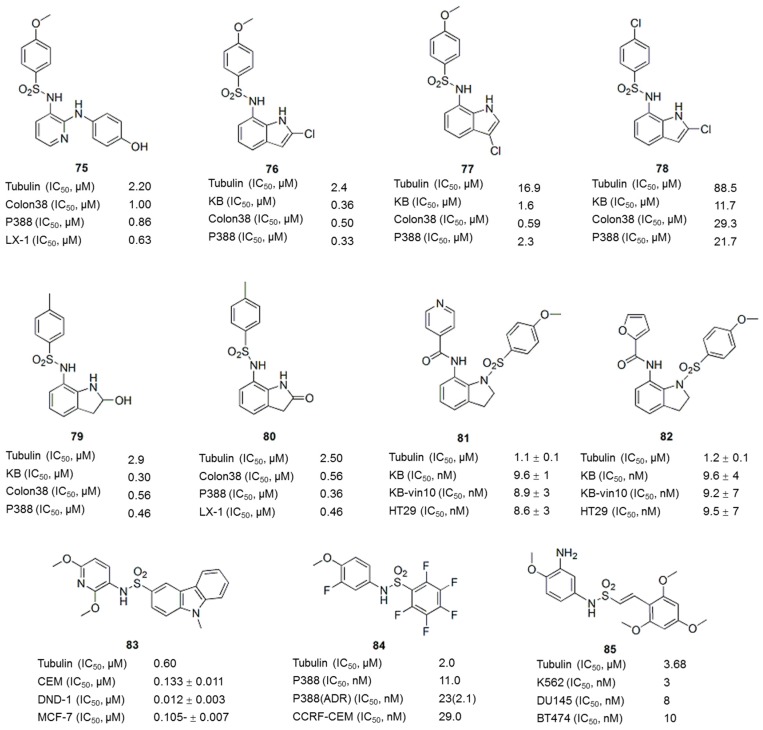
Sulfonamides.

Sulfonamides possess many types of biological activities, such as antibacterial, diuretic, antidiabetic, antithyroid, antihypertensive, or antiviral activities, and have been used in clinical settings [103]. The observation that many novel sulfonamide derivatives exhibit substantial antitumor activity is particularly interesting [104,105,106]. Since the discovery of E7010 (**75**, Figure 10) in 1992, sulfonamides targeting tubulin have emerged as an important class of anticancer agents [107]. E7010 (**75**), also known as ABT-751, is an orally bioavailable sulfonamide that inhibits tubulin polymerization by binding to the colchicine site on β-tubulin. E7010 exhibited a broad spectrum of antitumor activity in vitro and in vivo [108,109] and has entered Phase II clinical trials [110]. A series of compounds were identified from sulfonamide-focused libraries based on the structure of E7010 (**76**–**80** (Figure 10) [111,112], **81**, **82** (Figure 10) [108,111,113], **83** (Figure 10) [114,115]). These compounds inhibited tubulin polymerization and induced G_2_/M arrest, similar to E7010 (**75**). Medina et al. reported a series of pentafluorobenzenesulfonamide derivatives that displayed potent antiproliferative activity against a variety of tumor cells lines, including MDR tumor cell lines [116], with the compound T138067 (**84**) (Figure 10) showing the best activity. Interestingly, T138067 (**84**) (Figure 10) disrupted tubulin polymerization by covalently modifying β-tubulin at Cys239 [117]. Recently, some novel sulfonamide-based compounds have been shown to exhibit antiproliferative activity in different human cancer cell lines, with typical characteristics of tubulin-interacting agents [118,119,120,121]. For example, 2-(*N*-(3-chlorophenyl)-4-methoxyphenylsulfonamido)-*N*-hydroxypropan-amide (MPSP-001) inhibited the growth of 12 different types of human cancer cells (IC_50_ values ranging from 1.9 to 15.7 μM) and tubulin polymerization by directly binding to the colchicine site of β-tubulin [119]. A screen of the anticancer activity of twenty-four compounds containing the sulfonamide scaffold led to the identification of a naphthalene sulfonamide that acts as a novel reversible antimitotic agent by interfering with tubulin polymerization, which inhibited cancer cell motility and the growth of a representative panel of human cancer cell lines, with IC_50_ values in the nanomolar-to-low micromolar range [120]. In addition, some novel (*E*)-*N*-aryl-2-arylethene-sulfonamides showed potent cytotoxicity against a wide spectrum of cancer cell lines (IC_50_ values ranging from 5 to 10 nM) by disrupting tubulin assembly [121]. Among these compounds, compound **85** (Figure 10), the most potent compound, exhibits increased blood-brain barrier permeability compared to many clinically used antimitotic agents and inhibited tumor growth in vivo. Molecules with an aryl sulfonamide moiety containing 3-hydroxy, 4-methoxy groups or 3-amino, 4-methoxy groups and a styryl ring with methoxy groups at the 2, 4, and 6 positions showed optimum biological activity in SAR studies. Furthermore, the dimethyl quinone ester, water-soluble disodium phosphate or glycine analogue of compound **85** (Figure 10) showed good activity.

## 9. Thiazolidinone, Thiazole and Imidazole Analogues

### 9.1. Thiazolidinone Analogues

**Figure 11:**
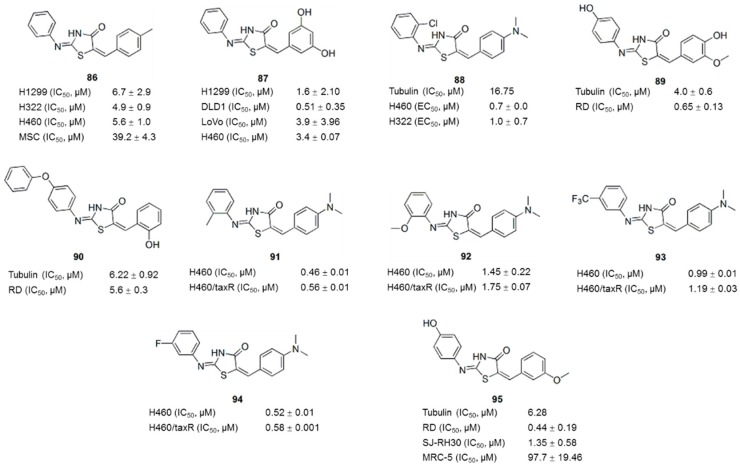
Thiazolidinone analogues.

**Figure 12:**
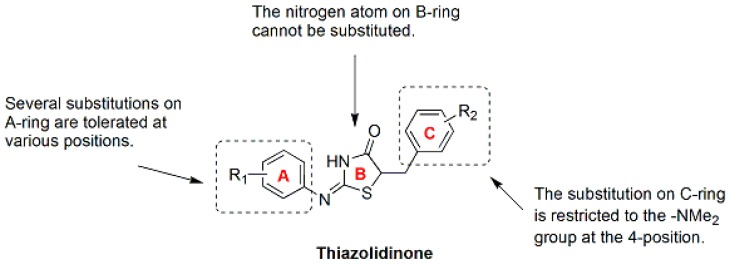
SAR of Thiazolidinone [128].

Thiazolidine is an important scaffold known to be associated with broad pharmacological activities in medicinal chemistry [122,123]. 4-thiazolidinones are derivatives of thiazolidine with a carbonyl group at the 4-position [122]. In a random screen of commercial compounds, compounds **86** and **87** (Figure 11), which contain a 4-thiazolidinone core structure, selectively killed drug-resistant cancer cells overexpressing P-gp by inducing apoptosis [124,125,126,127]. An iterative focused thiazolidinone library containing 372 compounds were synthesized and screened to explore the optimal pharmacophore structure and SAR for cytoselective toxicity and to improve the therapeutic window [128]. As a result, 10 potent cytoselective anticancer agents that selectively killed the paclitaxel-sensitive and paclitaxel-resistant non-small cell lung cancer (NSCLC) cell lines H460 and H460_TaxR_, respectively, but not normal cells, were identified. IC_50_ values for H460 and H460_TaxR_ cells were all <2.93 μM, and the IC_50_ values for normal NHFB cells were all >100 μM [128], indicating that these compounds are more effective but less toxic than previously reported compounds [125]. As shown in the SAR (Figure 12) studies, (a) the nitrogen atom on the 4-thiazolidinone ring cannot be substituted; (b) several substitutions on the A-ring are tolerated at various positions; and (c) the substitution on the C-ring is restricted to the -NMe_2_ group at the 4 position. A pharmacophore derived from active molecules suggested that two hydrogen bond acceptors and three hydrophobic regions were common features [128]. These active compounds exhibited anticancer activities across a wide variety of cancer cell lines, independent of P-gp expression, in further in vitro and in vivo studies [129,130,131,132,133,134]. As shown in mechanism studies, the inhibition of tubulin polymerization is one of the anticancer mechanisms of these active thiazolidinone compounds **88**–**95** (Figure 11).

Clinically available tubulin inhibitors, such as paclitaxel, vincristine, and colchicine, exhibit severe toxicity toward normal cells. Thus, in addition to the tubulin-targeting activity, other targets involved in the synergistic inhibition of cancer cells by thiazolidinone compounds and the mechanism underlying the reduced toxicity toward normal cells/organs have also been investigated. HSP90 has been shown to be another target of compound **88** (Figure 11) [131,132], whereas compound **89** (Figure 11) inhibited Dyrk1B kinase activity, which accounts for its 10-fold greater potency in the inhibition of rhabdomyosarcoma (RD) compared with compound **90** [135]. Interestingly, several active compounds **91**–**94** (Figure 11) were discovered to be histone deacetylase (HDAC) inhibitors [130], which accounts for their low toxicity [130,136,137] and synergistic anticancer effects [130,138,139]. Recently, the aqueous solubility (5-fold improvement in solubility, from 0.1 to 0.5 μg/mL) and anti-cancer activity (10-fold improvement in EC_50_ from 0.72–0.98 μM to 0.08–0.16 μM) of the new lead thiazolidinone compound were improved through all-around modifications, consecutive library synthesis, and testing [140]. In conclusion, thiazolidinone analogues have the potential for further development as a selective anticancer drug with low toxicity.

### 9.2. Thiazole and Imidazols Analogues

**Figure 13:**
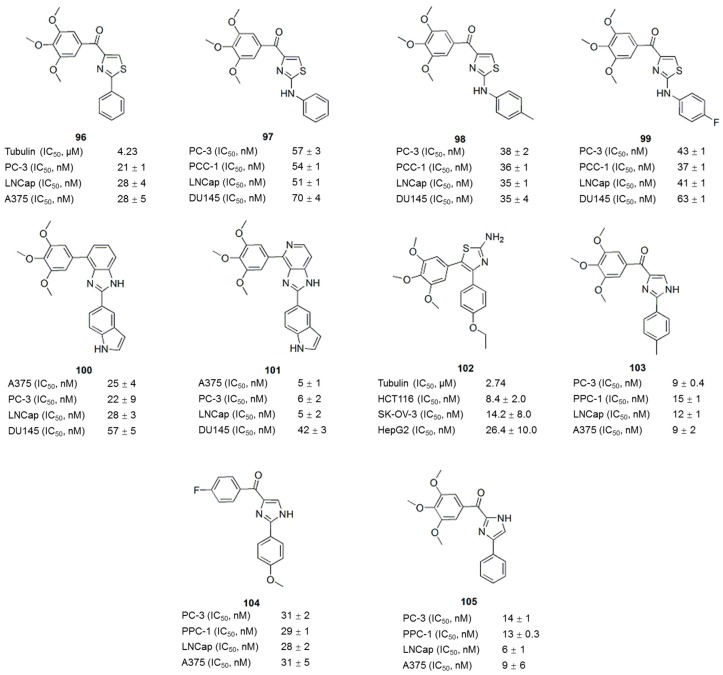
Thiazole and imidazole analogues.

**Figure 14:**
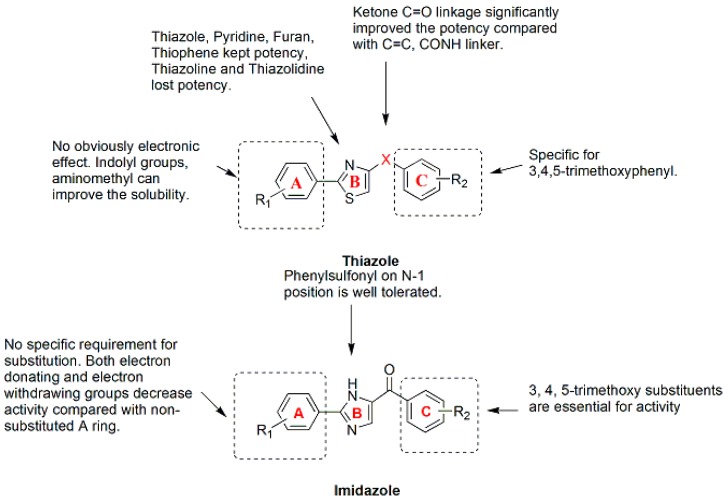
SAR of thiazole and imidazole analogues.

A series of 4-substituted methoxybenzoyl-aryl-thiazoles (SMART) based on the lead compound 2-arylthiazolidine-4-carboxylic acid amides (ATCAA) have been synthesized [141]. Of these SMART compounds, compound **96** (Figure 13) is a representative analogue that possesses nanomolar activity in inhibiting melanoma and prostate cancer cell growth in vitro. Based on preliminary studies, SMART compounds inhibit tubulin polymerization, effectively prevent the formation of functional microtubules and block cell mitosis. Furthermore, SMART compounds also showed great promise in overcoming Pgp mediated MDR in vitro [142]. Introducing an amino group between the A- and B-rings creates phenyl-aminothiazole (PAT); among these compounds, analogues **97**, **98** and **99** (Figure 13) showed similar antiproliferative potency against cancer cells, including MDR cancer cell lines. However, the solubility and in vivo bioavailability were greatly improved compared with the SMART compounds [143]. New derivatives with modifications at the carbonyl linker of SMART and modification of the A-ring of the PAT template have been synthesized To block the metabolically labile sites on **96** (Figure 13) [144]. The SAR analysis (Figure 14) of these compounds led to the identification of new benzimidazole and imidazo[4,5-*c*]pyridine-fused ring templates, represented by compounds **100** and **101** (Figure 13), respectively. Both compounds showed enhanced anticancer activity and substantially improved metabolic stability in liver microsomes compared to compound **96** and stronger inhibition of tubulin polymerization compared to colchicine. In addition, a series of 4,5-diarylthiazole derivatives were synthesized based on chemical modification of CA-4 through the transformation of N and S atom in the thiazole ring, and some new microtubule-destabilizing agents were identified [145]. Compound **102** (Figure 13), which contains a 3,4,5-trimethoxyphenyl group at the C-4 position and 4-ethoxyphenyl group at the C-5 position of the 2-amino substituted thiazole, was the most potent compound against five human cancer cell lines (IC_50_ values of 8.4–26.4 nM) in the SAR study. Compound **102** also circumvents drug resistance, possesses high antivascular activity in vitro and significantly suppresses tumor growth in xenograft models, indicating that compound **102** is a potent microtubule-destabilizing and vascular-disrupting agent for use in cancer treatment.

The thiazole ring of the SMART compounds was replaced with an imidazole to generate aryl-benzoylimidazoles (ABI), thus further improving the stability and hydrophilicity of these compounds [14]. The antiproliferative activities of these compounds were assessed in three melanoma cell lines and four human prostate cancer cell lines in vitro. The average IC_50_ of the most active compound (**103**) (Figure 13) was 15.7 nM. As revealed in mechanistic studies, the ABI analogues inhibit tubulin polymerization by interacting with the colchicine binding site. The ABI compounds exhibit substantially improved aqueous solubility and are equally potent against MDR cancer cells and the drug-sensitive parental melanoma cancer cells. Compound **104** (Figure 13) was more effective than dacarbazine (DTIC) in inhibiting melanoma xenograft tumor growth in vivo. Based on a rational structural modification of previous ABI analogues, a series of 4-aryl-2-benzoyl-imidazoles, named reverse ABI (RABI) analogues, were designed and synthesized [146]. Several RABI compounds exhibited excellent antiproliferative properties in vitro, with IC_50_ values in the low nanomolar range against eight cancer cell lines including MDR cancer cell lines. Among these compounds, compound **105** (Figure 13) was the most potent analogue, with an average IC_50_ of 14 nM. In addition, these new RABI analogues maintain their mechanisms of action by disrupting tubulin polymerization, similar to the ABI analogues (Figure 14). The SAR analysis of another new series of 4-aryl-5-(3,4,5-trimethoxyphenyl)-2-alkylthio-1*H*-imidazoles indicated that the presence of a 3,4,5-trimethoxy- phenyl moiety on the A-ring and 4-methoxy substituent on the B-ring had a beneficial effect on the cytotoxicity, which was related to the inhibition of microtubule polymerization [147].

## 10. Conclusions

Antitubulin agents are an important class of anticancer agents. Many antitubulin agents are derived from natural products and contain various heteroaromatic structures. Substantial efforts have been devoted to the development of new antitubulin agents by preparing analogues of the natural products or by synthesizing small molecules based on some privileged structures. Natural product- and privileged structure-promoted synthesis has been shown to be effective in lead compound discovery and optimization through the rapid generation of potential candidates for screening in vitro, thereby accelerating the discovery of novel therapeutic agents. Novel lead compounds or agents can overcome the limitations of existing tubulin inhibitors by improving the therapeutic window, the activity against MDR cancer cell lines, and the physical or pharmacological properties, such as aqueous solubility or pharmacokinetics. High throughput screening, molecular docking and SAR analyses will continue to play important roles in the discovery of antitubulin agents and drug discovery in general.

## References

[1] Wilson L., Jordan M.A. (1995). Microtubule dynamics: Taking aim at a moving target. Chem. Biol..

[2] Dumontet C., Jordan M.A. (2010). Microtubule-binding agents: A dynamic field of cancer therapeutics. Nat. Rev. Drug Discov..

[3] Jordan M.A., Wilson L. (2004). Microtubules as a target for anticancer drugs. Nat. Rev. Cancer.

[4] Fojo T., Menefee M. (2007). Mechanisms of multidrug resistance: The potential role of microtubule-stabilizing agents. Ann. Oncol..

[5] Buey R.M., Barasoain I., Jackson E., Meyer A., Giannakakou P., Paterson I., Mooberry S., Andreu J.M., Díaz J.F. (2005). Microtubule interactions with chemically diverse stabilizing agents: Thermodynamics of binding to the paclitaxel site predicts cytotoxicity. Chem. Biol..

[6] Jordan M.A., Thrower D., Wilson L. (1991). Mechanism of inhibition of cell proliferation by vinca alkaloids. Cancer Res..

[7] Jordan M.A., Wendell K., Gardiner S., Derry W.B., Copp H., Wilson L. (1996). Mitotic block induced in Hela cells by low concentrations of paclitaxel (taxol) results in abnormal mitotic exit and apoptotic cell death. Cancer Res..

[8] Brancale A., Silvestri R. (2007). Indole, a core nucleus for potent inhibitors of tubulin polymerization. Med. Res. Rev..

[9] Flynn B.L., Hamel E., Jung M.K. (2002). One-pot synthesis of benzo (b) furan and indole inhibitors of tubulin polymerization. J. Med. Chem..

[10] Flynn B.L., Gill G.S., Grobelny D.W., Chaplin J.H., Paul D., Leske A.F., Lavranos T.C., Chalmers D.K., Charman S.A., Kostewicz E. (2011). Discovery of 7-hydroxy-6-methoxy-2-methyl-3-(3,4,5-trimethoxybenzoyl)benzo (b) furan (BNC105), a tubulin polymerization inhibitor with potent antiproliferative and tumor vascular disrupting properties. J. Med. Chem..

[11] Ohsumi K., Hatanaka T., Fujita K., Nakagawa R., Fukuda Y., Nihei Y., Suga Y., Morinaga Y., Akiyama Y., Tsuji T. (1998). Syntheses and antitumor activity of *cis*-restricted combretastatins: 5-Membered heterocyclic analogues. Bioorg. Med. Chem. Lett..

[12] Banimustafa M., Kheirollahi A., Safavi M., Ardestani S.K., Aryapour H., Foroumadi A., Emami S. (2013). Synthesis and biological evaluation of 3-(trimethoxyphenyl)-2(3*H*)-thiazole thiones as combretastatin analogs. Eur. J. Med. Chem..

[13] Wang L., Woods K.W., Li Q., Barr K.J., McCroskey R.W., Hannick S.M., Gherke L., Credo R.B., Hui Y.H., Marsh K. (2002). Potent, orally active heterocycle-based combretastatin A-4 analogues: Synthesis, structure-activity relationship, pharmacokinetics, and in vivo antitumor activity evaluation. J. Med. Chem..

[14] Chen J.J., Wang Z., Li C.M., Lu Y., Vaddady P.K., Meibohm B., Dalton J.T., Miller D.D., Li W. (2010). Discovery of novel 2-aryl-4-benzoyl-imidazoles targeting the colchicines binding site in tubulin as potential anticancer agents. J. Med. Chem..

[15] Chaudhary V., Venghateri J.B., Dhaked H.P.S., Bhoyar A.S., Guchhait S.K., Panda D. (2016). Novel combretastatin-2-aminoimidazole analogues as potent tubulin assembly inhibitors: Exploration of unique pharmacophoric impact of bridging skeleton and aryl moiety. J. Med. Chem..

[16] Lu Y., Chen J.J., Xiao M., Li W., Miller D.D. (2012). An overview of tubulin inhibitors that interact with the colchicine binding site. Pharm. Res..

[17] Finkelstein Y., Aks S.E., Hutson J.R., Juurlink D.N., Nguyen P., Dubnov-Raz G., Pollak U., Koren G., Bentur Y. (2010). Colchicine poisoning: The dark side of an ancient drug. Clin. Toxicol..

[18] Quinn F.R., Neiman Z., Beisler J.A. (1981). Toxicity quantitative structure-activity relationships of colchicines. J. Med. Chem..

[19] Ringel I., Jaffe D., Alerhand S., Boye O., Muzaffar A., Brossi A. (1991). Fluorinated colchicinoids: Antitubulin and cytotoxic properties. J. Med. Chem..

[20] Das L., Datta A.B., Gupta S., Poddar A., Sengupta S., Janik M.E., Bhattacharyya B. (2005). -NH-dansyl isocolchicine exhibits a significantly improved tubulin-binding affinity and microtubule inhibition in comparison to isocolchicine by binding tubulin through its A and B rings. Biochemistry.

[21] Sapra S., Bhalla Y., Nandani Sharma S., Singh G., Nepali K., Budhiraja A., Dhar K.L. (2013). Colchicine and its various physicochemical and biological aspects. Med. Chem. Res..

[22] Tang-Wai D.F., Brossi A., Arnold L.D., Gros P. (1993). The nitrogen of the acetamido group of colchicine modulates P-glycoprotein-mediated multidrug resistance. Biochemistry.

[23] Cifuentes M., Schilling B., Ravindra R., Winter J., Janik M.E. (2006). Synthesis and biological evaluation of B-ring modified colchicine and isocolchicine analogs. Bioorgan. Med. Chem. Lett..

[24] Thomopoulou P., Sachs J., Teusch N., Mariappan A., Gopalakrishnan J., Schmalz H.-G.N. (2015). New colchicine-derived triazoles and their influence on cytotoxicity and microtubule morphology. ACS Med. Chem. Lett..

[25] Cosentino L., Redondo-Horcajo M., Zhao Y., Santos A.R., Chowdury K.F., Vinader V., Abdallah Q.M.A., Abdel-Rahman H., Fournier-Dit-Chabert J., Shnyder S.D. (2012). Synthesis and biological evaluation of colchicine B-ring analogues tethered with halogenated benzyl moieties. J. Med. Chem..

[26] Fournier-Dit-Chabert J., Vinader V., Santos A.R., Redondo-Horcajo M., Dreneau A., Basak R., Cosentino L., Marston G., Abdel-Rahman H., Loadman P.M. (2012). Synthesis and biological evaluation of colchicine C-ring analogues tethered with aliphatic linkers suitable for prodrug derivatisation. Bioorg. Med. Chem. Lett..

[27] Zefirova O.N., Nurieva E.V., Shishov D.V., Baskin I.I., Fuchs F., Lemcke H., Schroder F., Weiss D.G., Zefirov N.S., Kuznetsov S.A. (2011). Synthesis and SAR requirements of adamantane-colchicine conjugates with both microtubule depolymerizing and tubulin clustering activities. Bioorg. Med. Chem..

[28] Zefirova O.N., Lemcke H., Lantow M., Nurieva E.V., Wobith B., Onishchenko G.E., Hoenen A., Griffiths G., Zefirov N.S., Kuznetsov S.A. (2013). Unusual tubulin-clustering ability of specifically C7-modified colchicine analogues. ChemBioChem.

[29] Singh B., Kumar A., Joshi P., Guru S.K., Kumar S., Wani Z.A., Mahajan G., Hussain A., Qazi A.K., Kumar A. (2015). Colchicine derivatives with potent anticancer activity and reduced P-glycoprotein induction liability. Org. Biomol. Chem..

[30] Huczynski A., Rutkowski J., Popiel K., Maj E., Wietrzyk J., Stefanska J., Majcher U., Bartl F. (2015). Synthesis, antiproliferative and antibacterial evaluation of C-ring modified colchicine analogues. Eur. J. Med. Chem..

[31] Vilanova C., Diaz-Oltra S., Murga J., Falomir E., Carda M., Redondo-Horcajo M., Diaz J.F., Barasoain I., Marco J.A. (2014). Design and synthesis of pironetin analogue/colchicine hybrids and study of their cytotoxic activity and mechanisms of interaction with tubulin concepcion. J. Med. Chem..

[32] Lin C.M., Singh S.B., Chu P.S., Dempcy R.O., Schmidt J.M., Pettit G.R., Hamel E. (1988). Interactions of tubulin with potent natural and synthetic analogs of the antimitotic agent combretastatin: A structure-activity study. Mol. Pharmacol..

[33] Nam N.H. (2003). Combretastatin A-4 analogues as antimitotic antitumor agents. Curr. Med. Chem..

[34] Lee L., Davis R., Vanderham J., Hills P., Mackay H., Brown T., Mooberry S.L., Lee M. (2008). 1,2,3,4-tetrahydro-2-thioxopyrimidine analogs of combretastatin-A4. Eur. J. Med. Chem..

[35] Pettit G.R., Rhodes M.R., Herald D.L., Hamel E., Schmidt J.M., Pettitt R.K. (2005). Antineoplastic agents. 445. Synthesis and evaluation of structural modifications of (*Z*)- and (*E*)-combretastatin A-4. J. Med. Chem..

[36] Tron G.C., Pirali T., Sorba G., Pagliai F., Busacca S., Genazzani A.A. (2006). Medicinal chemistry of combretastatin A4: Present and future directions. J. Med. Chem..

[37] Alloatti D., Giannini G., Cabri W., Lustrati I., Marzi M., Ciacci A., Gallo G., Tinti M.O., Marcellini M., Riccioni T. (2008). Synthesis and biological activity of fluorinated combretastatin analogues. J. Med. Chem..

[38] Nagaiah G., Remick S.C. (2010). Combretastatin A4 phosphate: A novel vascular disrupting agent. Future Oncol..

[39] Ng Q.S., Mandeville H., Goh V., Alonzi R., Milner J., Carnell D., Meer K., Padhani A.R., Saunders M.I., Hoskin P.J. (2012). Phase Ib trial of radiotherapy in combination with combretastatin-A4-phosphate in patients with non-small-cell lung cancer, prostate adenocarcinoma, and squamous cell carcinoma of the head and neck. Ann. Oncol..

[40] Delmonte A., Sessa C. (2009). Ave8062: A new combretastatin derivative vascular disrupting agent. Expert Opin. Investig. Drugs.

[41] Lippert J.W. (2007). Vascular disrupting agents. Bioorg. Med. Chem..

[42] Pettit G.R., Lippert J.W. (2000). Antineoplastic agents 429. Syntheses of the combretastatin A-1 and combretastatin B-1 prodrugs. Anti-Cancer Drug Des..

[43] Devkota L., Lin C.M., Strecker T.E., Wang Y.F., Tidmore J.K., Chen Z., Guddneppanavar R., Jelinek C.J., Lopez R., Liu L. (2016). Design, synthesis, and biological evaluation of water-soluble amino acid prodrug conjugates derived from combretastatin, dihydronaphthalene, and benzosuberene-based parent vascular disrupting agents. Bioorg. Med. Chem..

[44] Duan Y.T., Man R.J., Tang D.J., Yao Y.F., Tao X.X., Yu C., Liang X.Y., Makawana J.A., Zou M.J., Wang Z.C. (2016). Design, synthesis and antitumor activity of novel link-bridge and B-ring modified combretastatin A-4 (CA-4) analogues as potent antitubulin agents. Sci. Rep..

[45] Gerova M.S., Stateva S.R., Radonova E.M., Kalenderska R.B., Rusew R.I., Nikolova R.P., Chanev C.D., Shivachev B.L., Apostolova M.D., Petrov O.I. (2016). Combretastatin A-4 analogues with benzoxazolone scaffold: Synthesis, structure and biological activity. Eur. J. Med. Chem..

[46] Do C.V., Faouzi A., Barette C., Farce A., Fauvarque M.O., Colomb E., Catry L., Berthier-Vergnes O., Haftek M., Barret R. (2016). Synthesis and biological evaluation of thiophene and benzo b thiophene analogs of combretastatin A-4 and isocombretastatin A-4: A comparison between the linkage positions of the 3,4,5-trimethoxystyrene unit. Bioorg. Med. Chem. Lett..

[47] Guan Q., Zuo D.Y., Jiang N., Qi H., Zhai Y.P., Bai Z.S., Feng D.J., Yang L., Jiang M.Y., Bao K. (2015). Microwave-assisted synthesis and biological evaluation of 3,4-diaryl maleic anhydride/*n*-substituted maleimide derivatives as combretastatin A-4 analogues. Bioorg. Med. Chem. Lett..

[48] Pati H.N., Wicks M., Holt H.L., LeBlanc R., Weisbruch P., Forrest L., Lee M. (2005). Synthesis and biological evaluation of *cis*-combretastatin analogs and their novel 1,2,3-triazole derivatives. Heterocycl. Commun..

[49] Odlo K., Hentzen J., dit Chabert J.F., Ducki S., Gani O., Sylte I., Skrede M., Florenes V.A., Hansen T.V. (2008). 1,5-disubstituted 1,2,3-triazoles as *cis*-restricted analogues of combretastatin A-4: Synthesis, molecular modeling and evaluation as cytotoxic agents and inhibitors of tubulin. Bioorg. Med. Chem..

[50] Romagnoli R., Baraldi P.G., Salvador M.K., Preti D., Tabrizi M.A., Brancale A., Fu X.H., Li J., Zhang S.Z., Hamel E. (2012). Synthesis and evaluation of 1,5-disubstituted tetrazoles as rigid analogues of combretastatin A-4 with potent antiproliferative and antitumor activity. J. Med. Chem..

[51] Carr M., Greene L.M., Knox A.J.S., Lloyd D.G., Zisterer D.M., Meegan M.J. (2010). Lead identification of conformationally restricted β-lactam type combretastatin analogues: Synthesis, antiproliferative activity and tubulin targeting effects. Eur. J. Med. Chem..

[52] Guan Q., Yang F.S., Guo D.D., Xu J.W., Jiang M.Y., Liu C.J., Bao K., Wu Y.L., Zhang W.G. (2014). Synthesis and biological evaluation of novel 3,4-diaryl-1,2,5-selenadiazol analogues of combretastatin A-4. Eur. J. Med. Chem..

[53] Wang Z., Yang Q.K., Bai Z.S., Sun J., Jiang X.W., Song H.R., Wu Y.L., Zhang W.G. (2015). Synthesis and biological evaluation of 2,3-diarylthiophene analogues of combretastatin A-4. MedChemComm.

[54] Sanghai N., Jain V., Preet R., Kandekar S., Das S., Trivedi N., Mohapatra P., Priyadarshani G., Kashyap M., Das D. (2014). Combretastatin A-4 inspired novel 2-aryl-3-arylamino-imidazo-pyridines/pyrazines as tubulin polymerization inhibitors, antimitotic and anticancer agents. MedChemComm.

[55] Hu J.H., Yan J., Chen J., Pang Y.Q., Huang L., Li X.S. (2015). Synthesis, biological evaluation and mechanism study of a class of cyclic combretastatin A-4 analogues as novel antitumour agents. MedChemComm.

[56] Chen H., Li Y., Sheng C., Lv Z., Dong G., Wang T., Liu J., Zhang M., Li L., Zhang T. (2013). Design and synthesis of cyclopropylamide analogues of combretastatin-A4 as novel microtubule-stabilizing agents. J. Med. Chem..

[57] Zheng S.L., Zhong Q., Mottamal M., Zhang Q., Zhang C.D., LeMelle E., McFerrin H., Wang G.D. (2014). Design, synthesis, and biological evaluation of novel pyridine-bridged analogues of combretastatin-A4 as anticancer agents. J. Med. Chem..

[58] Kaushik N.K., Kaushik N., Attri P., Kumar N., Kim C.H., Verma A.K., Choi E.H. (2013). Biomedical importance of indoles. Molecules.

[59] Alves F.R.D., Barreiro E.J., Fraga C.A.M. (2009). From nature to drug discovery: The indole scaffold as a “privileged structure”. Mini-Rev. Med. Chem..

[60] Patil S.A., Patil R., Miller D.D. (2012). Indole molecules as inhibitors of tubulin polymerization: Potential new anticancer agents. Future Med. Chem..

[61] Giansanti V., Piscitelli F., Camboni T., Prosperi E., La Regina G., Parks M., Silvestri R., Scovassi A.I. (2010). Arylthioindoles: Promising compounds against cancer cell proliferation. Oncol. Lett..

[62] De Martino G., La Regina G., Coluccia A., Edler M.C., Barbera M.C., Brancale A., Wilcox E., Hamel E., Artico M., Silvestri R. (2004). Arylthioindoles, potent inhibitors of tubulin polymerization. J. Med. Chem..

[63] De Martino G., Edler M.C., La Regina G., Coluccia A., Barbera M.C., Barrow D., Nicholson R.I., Chiosis G., Brancale A., Hamel E. (2006). New arylthioindoles: Potent inhibitors of tubulin polymerization. 2. Structure-activity relationships and molecular modeling studies. J. Med. Chem..

[64] La Regina G., Edler M.C., Brancale A., Kandil S., Coluccia A., Piscitelli F., Hamel E., De Martino G., Matesanz R., Diaz J.F. (2007). Arylthioindole inhibitors of tubulin polymerization. 3. Biological evaluation, structure-activity relationships and molecular modeling studies. J. Med. Chem..

[65] La Regina G., Sarkar T., Bai R.L., Edler M.C., Saletti R., Coluccia A., Piscitelli F., Minelli L., Gatti V., Mazzoccoli C. (2009). New arylthioindoles and related bioisosteres at the sulfur bridging group. 4. Synthesis, tubulin polymerization, cell growth inhibition, and molecular modeling studies. J. Med. Chem..

[66] La Regina G., Bai R., Rensen W., Coluccia A., Piscitelli F., Gatti V., Bolognesi A., Lavecchia A., Granata I., Porta A. (2011). Design and synthesis of 2-heterocyclyl-3-arylthio-1*H*-indoles as potent tubulin polymerization and cell growth inhibitors with improved metabolic stability. J. Med. Chem..

[67] La Regina G., Bai R.L., Rensen W.M., Di Cesare E., Coluccia A., Piscitelli F., Famiglini V., Reggio A., Nalli M., Pelliccia S. (2013). Toward highly potent cancer agents by modulating the C-2 group of the arylthioindole class of tubulin polymerization inhibitors. J. Med. Chem..

[68] Colley H.E., Muthana M., Danson S.J., Jackson L.V., Brett M.L., Harrison J., Coole S.F., Mason D.P., Jennings L.R., Wong M. (2015). An orally bioavailable, indole-3-glyoxylamide based series of tubulin polymerization inhibitors showing tumor growth inhibition in a mouse xenograft model of head and neck cancer. J. Med. Chem..

[69] MacDonough M.T., Strecker T.E., Hamel E., Hall J.J., Chaplin D.J., Trawick M.L., Pinney K.G. (2013). Synthesis and biological evaluation of indole-based, anti-cancer agents inspired by the vascular disrupting agent 2-(3′-hydroxy-4′-methoxyphenyl)-3-(3″,4″,5″-trimethoxybenzoyl)-6-methoxyindole (oxi8006). Bioorgan. Med. Chem..

[70] La Regina G., Bai R.L., Coluccia A., Farniglini V., Pelliccia S., Passacantilli S., Mazzoccoli C., Ruggieri V., Verrico A., Miele A. (2015). New indole tubulin assembly inhibitors cause stable arrest of mitotic progression, enhanced stimulation of natural killer cell cytotoxic activity, and repression of hedgehog-dependent cancer. J. Med. Chem..

[71] Wen Z.Y., Xu J.W., Wang Z.W., Qi H., Xu Q.L., Bai Z.S., Zhang Q., Bao K., Wu Y.L., Zhang W.G. (2015). 3-(3,4,5-trimethoxyphenylselenyl)-1*H*-indoles and their selenoxides as combretastatin A-4 analogs: Microwave-assisted synthesis and biological evaluation. Eur. J. Med. Chem..

[72] Kamal A., Reddy V.S., Karnewar S., Chourasiya S.S., Shaik A.B., Kumar G.B., Kishor C., Reddy M.K., Narasimha Rao M., Nagabhushana A. (2013). Synthesis and biological evaluation of imidazopyridine-oxindole conjugates as microtubule-targeting agents. ChemMedChem.

[73] Hwang D.J., Wang J., Li W., Miller D.D. (2015). Structural optimization of indole derivatives acting at colchicine binding site as potential anticancer agents. ACS Med. Chem. Lett..

[74] Hu M.J., Zhang B., Yang H.K., Liu Y., Chen Y.R., Ma T.Z., Lu L., You W.W., Zhao P.L. (2015). Design, synthesis and molecular docking studies of novel indole-pyrimidine hybrids as tubulin polymerization inhibitors. Chem. Biol. Drug Des..

[75] Orlikova B., Tasdemir D., Golais F., Dicato M., Diederich M. (2011). Dietary chalcones with chemopreventive and chemotherapeutic potential. Genes Nutr..

[76] Mahapatra D.M., Bharti S.K., Asati V. (2015). Anti-cancer chalcones: Structural and molecular target perspectives. Eur. J. Med. Chem..

[77] Lawrence N.J., Rennison D., McGown A.T., Ducki S., Gul L.A., Hadfield J.A., Khan N. (2001). Linked parallel synthesis and MTT bioassay screening of substituted chalcones. J. Comb. Chem..

[78] Ducki S., Rennison D., Woo M., Kendall A., Chabert J.F.D., McGown A.T., Lawrence N.J. (2009). Combretastatin-like chalcones as inhibitors of microtubule polymerization. Part 1: Synthesis and biological evaluation of antivascular activity. Bioorg. Med. Chem..

[79] Qian Y., Ma G.Y., Yang Y., Cheng K., Zheng Q.Z., Mao W.J., Shi L., Zhao J., Zhu H.L. (2010). Synthesis, molecular modeling and biological evaluation of dithiocarbamates as novel antitubulin agents. Bioorg. Med. Chem..

[80] Salum L.B., Altei W.F., Chiaradia L.D., Cordeiro M.N.S., Canevarolo R.R., Melo C.P.S., Winter E., Mattei B., Daghestani H.N., Santos-Silva M.C. (2013). Cytotoxic 3,4,5-trimethoxychalcones as mitotic arresters and cell migration inhibitors. Eur. J. Med. Chem..

[81] Wang Y.T., Qin Y.J., Zhang Y.L., Li Y.J., Rao B., Zhang Y.Q., Yang M.R., Jiang A.Q., Qi J.L., Zhu H.L. (2014). Synthesis, biological evaluation, and molecular docking studies of novel chalcone oxime derivatives as potential tubulin polymerization inhibitors. RSC Adv..

[82] Zhu C.G., Zuo Y.L., Wang R.M., Liang B.X., Yue X., Wen G.S., Shang N.N., Huang L., Chen Y., Du J. (2014). Discovery of potent cytotoxic ortho-aryl chalcones as new scaffold targeting tubulin and mitosis with affinity-based fluorescence. J. Med. Chem..

[83] Wang G.C., Li C.Y., He L., Lei K., Wang F., Pu Y.Z., Yang Z., Cao D., Ma L., Chen J.Y. (2014). Design, synthesis and biological evaluation of a series of pyrano chalcone derivatives containing indole moiety as novel anti-tubulin agents. Bioorg. Med. Chem..

[84] Chen J., Yan J., Hu J.H., Pang Y.Q., Huang L., Li X.S. (2015). Synthesis, biological evaluation and mechanism study of chalcone analogues as novel anti-cancer agents. RSC Adv..

[85] Yan J., Chen J., Zhang S., Hu J.H., Huang L., Li X.S. (2016). Synthesis, evaluation, and mechanism study of novel indole-chalcone derivatives exerting effective antitumor activity through microtubule destabilization in vitro and in vivo. J. Med. Chem..

[86] Singh P., Kaur M., Holzer W. (2010). Synthesis and evaluation of indole, pyrazole, chromone and pyrimidine based conjugates for tumor growth inhibitory activities—Development of highly efficacious cytotoxic agents. Eur. J. Med. Chem..

[87] Kamal A., Balakrishna M., Nayak V.L., Shaik T.B., Faazil S., Nimbarte V.D. (2014). Design and synthesis of imidazo 2,1-B thiazole-chalcone conjugates: Microtubule-destabilizing agents. Mini-Rev. Med. Chem..

[88] Ruan B.F., Lu X.A., Tang J.F., Wei Y., Wang X.L., Zhang Y.B., Wang L.S., Zhu H.L. (2011). Synthesis, biological evaluation, and molecular docking studies of resveratrol derivatives possessing chalcone moiety as potential antitubulin agents. Bioorg. Med. Chem..

[89] Imbert T.F. (1998). Discovery of podophyllotoxins. Biochimie.

[90] Liu Y.Q., Tian J., Qian K., Zhao X.B., Morris-Natschke S.L., Yang L., Nan X., Tian X., Lee K.H. (2015). Recent progress on C-4-modified podophyllotoxin analogs as potent antitumor agents. Med. Res. Rev..

[91] Gordaliza M., Castro M.A., del Corral J.M.M., San Feliciano A. (2000). Antitumor properties of podophyllotoxin and related compounds. Curr. Pharm. Des..

[92] You Y.J. (2005). Podophyllotoxin derivatives: Current synthetic approaches for new anticancer agents. Curr. Pharm. Des..

[93] Abad A., Lopez-Perez J.L., del Olmo E., Garcia-Fernandez L.F., Francesch A., Trigili C., Barasoain I., Andreu J.M., Diaz J.F., San Feliciano A. (2012). Synthesis and antimitotic and tubulin interaction profiles of novel pinacol derivatives of podophyllotoxins. J. Med. Chem..

[94] Nepali K., Ojha R., Sharma S., Bedi P.M.S., Dhar K.L. (2014). Tubulin inhibitors: A patent survey. Recent Pat. Anti-Cancer Drug Discov..

[95] Liu Y.Q., Wei D.F., Zhao Y.L., Cheng W.D., Lu Y., Ma Y.Q., Li X., Han C., Wei Y.X., Cao H.M. (2012). Synthesis and biological evaluation of a series of podophyllotoxins derivatives as a class of potent antitubulin agents. Bioorg. Med. Chem..

[96] Labruere R., Gautier B., Testud M., Seguin J., Lenoir C., Desbene-Finck S., Helissey P., Garbay C., Chabot G.G., Vidal M. (2010). Design, synthesis, and biological evaluation of the first podophyllotoxin analogues as potential vascular-disrupting agents. Mini-Rev. Med. Chem..

[97] Rosania G.R., Chang Y.-T., Perez O., Sutherlin D., Dong H., Lockhart D.J., Schultz P.G. (2000). Myoseverin, a microtubule-binding molecule with novel cellular effects. Nat. Biotechnol..

[98] Moon H.-S., Jacobson E.M., Khersonsky S.M., Luzung M.R., Walsh D.P., Xiong W., Lee J.W., Parikh P.B., Lam J.C., Kang T.-W. (2002). A novel microtubule destabilizing entity from orthogonal synthesis of triazine library and zebrafish embryo screening. J. Am. Chem. Soc..

[99] Chang Y.-T., Wignall S.M., Rosania G.R., Gray N.S., Hanson S.R., Su A.I., Merlie J., Moon H.-S., Sangankar S.B., Perez O. (2001). Synthesis and biological evaluation of myoseverin derivatives: Microtubule assembly inhibitors. J. Med. Chem..

[100] Krystof V., Moravcova D., Paprskarova M., Barbier P., Peyrot V., Hlobilkova A., Havlicek L., Strnad M. (2006). Synthesis and biological activity of 8-azapurine and pyrazolo 4,3-*d* pyrimidine analogues of myoseverin. Eur. J. Med. Chem..

[101] Perez O.D., Chang Y.-T., Rosania G., Sutherlin D., Schultz P.G. (2002). Inhibition and reversal of myogenic differentiation by purine-based microtubule assembly inhibitors. Chem. Biol..

[102] Popowycz F., Schneider C., DeBonis S., Skoufias D.A., Kozielski F., Galmarini C.M., Joseph B. (2009). Synthesis and antiproliferative evaluation of pyrazolo[1,5-*a*]-1,3,5-triazine myoseverin derivatives. Bioorgan. Med. Chem..

[103] Drews J. (2000). Drug discovery: A historical perspective. Science.

[104] Scozzafava A., Owa T., Mastrolorenzo A., Supuran C.T. (2003). Anticancer and antiviral sulfonamides. Curr. Med. Chem..

[105] Banerjee M., Poddar A., Mitra G., Surolia A., Owa T., Bhattacharyya B. (2005). Sulfonamide drugs binding to the colchicine site of tubulin: Thermodynamic analysis of the drug-tubulin interactions by isothermal titration calorimetry. J. Med. Chem..

[106] Hu L.X., Li Z.R., Jiang J.D., Boykin D.W. (2008). Novel diaryl or heterocyclic sulfonamides as antimitotic agents. Anti-Cancer Agents Med. Chem..

[107] Yoshino H., Ueda N., Niijima J., Sugumi H., Kotake Y., Koyanagi N., Yoshimatsu K., Asada M., Watanabe T., Nagasu T. (1992). Novel sulfonamides as potential, systemically active antitumor agents. J. Med. Chem..

[108] Yamamoto K., Noda K., Yoshimura A., Fukuoka M., Furuse K., Niitani H. (1998). Phase I study of E7010. Cancer Chemother. Pharmacol..

[109] Hande K.R., Hagey A., Berlin J., Cai Y.N., Meek K., Kobayashi H., Lockhart A.C., Medina D., Sosman J., Gordon G.B. (2006). The pharmacokinetics and safety of ABT-751, a novel, orally bioavailable sulfonamide antimitotic agent: Results of a phase 1 study. Clin. Cancer Res..

[110] Mauer A.M., Cohen E.E.W., Ma P.C., Kozloff M.F., Schwartzberg L., Coates A.I., Qian J., Hagey A.E., Gordon G.B. (2008). A phase ii study of ABT-751 in patients with advanced non-small cell lung cancer. J. Thorac. Oncol..

[111] Owa T., Okauchi T., Yoshimatsu K., Sugi N.H., Ozawa Y., Nagasu T., Koyanagi N., Okabe T., Kitoh K., Yoshino H. (2000). A focused compound library of novel *n*-(7-indolyl)benzenesulfonamides for the discovery of potent cell cycle inhibitors. Bioorg. Med. Chem. Lett..

[112] Yokoi A., Kuromitsu J., Kawai T., Nagasu T., Sugi N.H., Yoshimatsu K., Yoshino H., Owa T. (2002). Profiling novel sulfonamide antitumor agents with cell-based phenotypic screens and array-based gene expression analysis. Mol. Cancer Ther..

[113] Chang J.Y., Hsieh H.P., Chang C.Y., Hsu K.S., Chiang Y.F., Chen C.M., Kuo C.C., Liou J.P. (2006). 7-aroyl-aminoindoline-1-sulfonamides as a novel class of potent antitubulin agents. J. Med. Chem..

[114] Hu L.X., Li Z.R., Wang Y.M., Wu Y.B., Jiang J.D., Boykin D.W. (2007). Novel pyridinyl and pyrimidinylcarbazole sulfonamides as antiproliferative agents. Bioorg. Med. Chem. Lett..

[115] Wang Y.M., Hu L.X., Liu Z.M., You X.F., Zhang S.H., Qu J.R., Li Z.R., Li Y., Kong W.J., He H.W. (2008). *N*-(2,6-dimethoxypyridine-3-yl)-9-methylcarbazole-3-sulfonamide as a novel tubulin ligand against human cancer. Clin. Cancer Res..

[116] Medina J.C., Shan B., Beckmann H., Farrell R.P., Clark D.L., Learned R.M., Roche D., Li A., Baichwal V., Case C. (1998). Novel antineoplastic agents with efficacy against multidrug resistant tumor cells. Bioorg. Med. Chem. Lett..

[117] Shan B., Medina J.C., Santha E., Frankmoelle W.P., Chou T.C., Learned R.M., Narbut M.R., Stott D., Wu P.G., Jaen J.C. (1999). Selective, covalent modification of β-tubulin residue CYS-239 by t138067, an antitumor agent with in vivo efficacy against multidrug-resistant tumors. Proc. Natl. Acad. Sci. USA.

[118] Abbassi N., Chicha H., Rakib E., Hannioui A., Alaoui M., Hajjaji A., Geffken D., Aiello C., Gangemi R., Rosano C. (2012). Synthesis, antiproliferative and apoptotic activities of *n*-(6(4)-indazolyl)-benzenesulfonamide derivatives as potential anticancer agents. Eur. J. Med. Chem..

[119] Liu Z.L., Tian W., Wang Y., Kuang S., Luo X.M., Yu Q. (2012). A novel sulfonamide agent, MPSP-001, exhibits potent activity against human cancer cells in vitro through disruption of microtubule. Acta Pharmacol. Sin..

[120] Aceves-Luquero C., Galiana-Roselló C., Ramis G., Villalonga-Planells R., García-España E., Fernández de Mattos S., Peláez R., Llinares J.M., González-Rosende M.E., Villalonga P. (2016). *N*-(2-methyl-indol-1*H*-5-yl)-1-naphthalenesulfonamide: A novel reversible antimitotic agent inhibiting cancer cell motility. Biochem. Pharmacol..

[121] Reddy M.V.R., Mallireddigari M.R., Pallela V.R., Cosenza S.C., Billa V.K., Akula B., Subbaiah D., Bharathi E.V., Padgaonkar A., Lv H. (2013). Design, synthesis, and biological evaluation of (*E*)-*n*-aryl-2-arylethenesulfonamide analogues as potent and orally bioavailable microtubule-targeted anticancer agents. J. Med. Chem..

[122] Verma A., Saraf S.K. (2008). 4-thiazolidinone—A biologically active scaffold. Eur. J. Med. Chem..

[123] Zhang Q., Zhou H.Y., Zhai S.M., Yan B. (2010). Natural product-inspired synthesis of thiazolidine and thiazolidinone compounds and their anticancer activities. Curr. Pharm. Des..

[124] Teraishi F., Wu S., Sasaki J., Zhang L., Davis J., Guo W., Dong F., Fang B. (2005). Jnk1-dependent antimitotic activity of thiazolidin compounds in human non-small-cell lung and colon cancer cells. Cell. Mol. Life Sci..

[125] Teraishi F., Wu S., Sasaki J., Zhang L., Zhu H.-B., Davis J.J., Fang B. (2005). P-glycoprotein-independent apoptosis induction by a novel synthetic compound, MMPT [5-[(4-methylphenyl) methylene]-2-(phenylamino)-4(5*H*)-thiazolone]. J. Pharmacol. Exp. Ther..

[126] Wu S., Guo W., Teraishi F., Pang J., Kaluarachchi K., Zhang L., Davis J., Dong F., Yan B., Fang B. (2006). Anticancer activity of 5-benzylidene-2-phenylimino-1, 3-thiazolidin-4-one (BPT) analogs. Med. Chem..

[127] Teraishi F., Wu S., Zhang L., Guo W., Davis J.J., Dong F., Fang B. (2005). Identification of a novel synthetic thiazolidin compound capable of inducing c-Jun NH_2_-terminal Kinase-Dependent apoptosis in human colon cancer cells. Cancer Res..

[128] Zhou H., Wu S., Zhai S., Liu A., Sun Y., Li R., Zhang Y., Ekins S., Swaan P.W., Fang B. (2008). Design, synthesis, cytoselective toxicity, structure-activity relationships, and pharmacophore of thiazolidinone derivatives targeting drug-resistant lung cancer cells. J. Med. Chem..

[129] Li L., Zhang Q., Liu A., Li X., Zhou H., Liu Y., Yan B. (2011). Proteome interrogation using nanoprobes to identify targets of a cancer-killing molecule. J. Am. Chem. Soc..

[130] Zhang C., Zhai S., Li X., Zhang Q., Wu L., Liu Y., Jiang C., Zhou H., Li F., Zhang S. (2014). Synergistic action by multi-targeting compounds produces a potent compound combination for human NSCLC both in vitro and in vivo. Cell Death Dis..

[131] Zhang Q., Zhai S., Li L., Li X., Zhou H., Liu A., Su G., Mu Q., Du Y., Yan B. (2013). Anti-tumor selectivity of a novel tubulin and HSP90 dual-targeting inhibitor in non-small cell lung cancer models. Biochem. Pharmacol..

[132] Zhang Q., Zhai S., Li L., Li X., Jiang C., Zhang C., Yan B. (2014). P-glycoprotein-evading anti-tumor activity of a novel tubulin and HSP90 dual inhibitor in a non-small-cell lung cancer model. J. Pharmacol. Sci..

[133] Mu Y., Liu Y., Li L., Tian C., Zhou H., Zhang Q., Yan B. (2015). The novel tubulin polymerization inhibitor mhpt exhibits selective anti-tumor activity against rhabdomyosarcoma in vitro and in vivo. PLoS ONE.

[134] Zhang Q., Liu X., Li X., Li C., Zhou H., Yan B. (2013). Antitumor activity of (2*E*,5*Z*)-5-(2-hydroxybenzylidene)-2-((4-phenoxyphenyl) imino) thiazolidin-4-one, a novel microtubule-depolymerizing agent, in U87MG human glioblastoma cells and corresponding mouse xenograft model. J. Pharmacol. Sci..

[135] Li L., Liu Y., Zhang Q., Zhou H., Zhang Y., Yan B. (2014). Comparison of cancer cell survival triggered by microtubule damage after turning DYRK1B kinase on and off. ACS Chem. Biol..

[136] Dokmanovic M., Clarke C., Marks P.A. (2007). Histone deacetylase inhibitors: Overview and perspectives. Mol. Cancer Res..

[137] Lee J.-H., Choy M., Ngo L., Foster S., Marks P.A. (2010). Histone deacetylase inhibitor induces DNA damage, which normal but not transformed cells can repair. Proc. Natl. Acad. Sci. USA.

[138] Dowdy S.C., Jiang S., Zhou X.C., Hou X., Jin F., Podratz K.C., Jiang S.-W. (2006). Histone deacetylase inhibitors and paclitaxel cause synergistic effects on apoptosis and microtubule stabilization in papillary serous endometrial cancer cells. Mol. Cancer Ther..

[139] Zuco V., de Cesare M., Cincinelli R., Nannei R., Pisano C., Zaffaroni N., Zunino F. (2011). Synergistic antitumor effects of novel HDAC inhibitors and paclitaxel in vitro and in vivo. PLoS ONE.

[140] Liu Y., Li F., Wu L., Wang W., Zhu H., Zhang Q., Zhou H., Yan B. (2015). Improving both aqueous solubility and anti-cancer activity by assessing progressive lead optimization libraries. Bioorg. Med. Chem. Lett..

[141] Lu Y., Li C.M., Wang Z., Ross C.R., Chen J., Dalton J.T., Li W., Miller D.D. (2009). Discovery of 4-substituted methoxybenzoyl-aryl-thiazole as novel anticancer agents: Synthesis, biological evaluation, and structure-activity relationships. J. Med. Chem..

[142] Li F., Lu Y., Li W., Miller D.D., Mahato R.I. (2010). Synthesis, formulation and in vitro evaluation of a novel microtubule destabilizer, smart-100. J. Control. Release.

[143] Lu Y., Li C.M., Wang Z., Chen J.J., Mohler M.L., Li W., Dalton J.T., Miller D.D. (2011). Design, synthesis, and SAR studies of 4-substituted methoxylbenzoyl-aryl-thiazoles analogues as potent and orally bioavailable anticancer agents. J. Med. Chem..

[144] Lu Y., Chen J.J., Wang J., Li C.M., Ahn S., Barrett C.M., Dalton J.T., Li W., Miller D.D. (2014). Design, synthesis, and biological evaluation of stable colchicine binding site tubulin inhibitors as potential anticancer agents. J. Med. Chem..

[145] Wang F., Yang Z., Liu Y.B., Ma L., Wu Y.Z., He L., Shao M.F., Yu K., Wu W.S., Pu Y.Z. (2015). Synthesis and biological evaluation of diarylthiazole derivatives as antimitotic and antivascular agents with potent antitumor activity. Bioorg. Med. Chem..

[146] Xiao M., Ahn S.J., Wang J., Chen J.J., Miller D.D., Dalton J.T., Li W. (2013). Discovery of 4-aryl-2-benzoyl-imidazoles as tubulin polymerization inhibitor with potent antiproliferative properties. J. Med. Chem..

[147] Assadieskandar A., Amini M., Ostad S.N., Riazi G.H., Cheraghi-Shavi T., Shafiei B., Shafiee A. (2013). Design, synthesis, cytotoxic evaluation and tubulin inhibitory activity of 4-aryl-5-(3,4,5-trimethoxyphenyl)-2-alkylthio-1*H*-imidazole derivatives. Bioorg. Med. Chem..

